# New insights on the shell-crusher shark *Ptychodus decurrens* Agassiz, 1838 (Elasmobranchii, Ptychodontidae) based on the first known articulated dentition from the Upper Cretaceous of Croatia

**DOI:** 10.1186/s13358-024-00340-7

**Published:** 2025-01-08

**Authors:** Manuel Amadori, Sanja Japundžić, Jacopo Amalfitano, Luca Giusberti, Eliana Fornaciari, Patrick L. Jambura, Jürgen Kriwet

**Affiliations:** 1 Department of Palaeontology, Geozentrum, University of Vienna, Josef-Holaubek-Platz 2, 1090 Vienna, Austria; 2 Croatian Natural History Museum, Demetrova Ul. 1, 10000 Zagreb, Croatia; 3 Dipartimento Di Geoscienze, Università degli Studi di Padova, Via Gradenigo 6, 35131 Padua, Italy; 4 Vienna Doctoral School of Ecology and Evolution (VDSEE), University of Vienna, Djerassiplatz 1, 1030 Vienna, Austria

**Keywords:** Tooth plate, Durophagy, Lamniformes, Taxonomy, Dalmatia, Turonian

## Abstract

A new lower tooth plate of *Ptychodus decurrens* from the Turonian (Upper Cretaceous) of the Dalmatian region (southern Croatia) is documented here for the first time. The specimen represents the first articulated dentition of a ptychodontid shark that has been discovered from the Balkan Peninsula and the most complete ever found for the un-cuspidate species *P. decurrens* up to now. The reconstruction of the entire lower dentition of *P. decurrens* based on this exceptionally well-preserved dentition shows a wider crushing plate than previously hypothesised with bulgy teeth limited to the central area. Even though a defined cusp is missing, the occlusal surface of the teeth is undeniably raised and bulgy in some un-cuspidate species of *Ptychodus* (e.g., *P. decurrens*). This compels us to reconsider the use of terms such as high- and low-crowned as well as cuspidate and un-cuspidate. In addition, specimens previously assigned to dubious species (*P. depressus*, *P. levis* and *P. oweni*) or even varieties (*P. polygyrus* var. *sulcatus* and *P. decurrens* var. *multiplicatus*) are reassigned here to *P. decurrens* based on a careful comparison of the type materials. The reassessment of tooth root morphologies provides indicative traits for the identification of different genera of ptychodontid sharks (*Paraptychodus* and *Ptychodus*). The taxonomic revision presented here is crucial for securing a stable taxonomy and systematics of the shell-crushing shark *P. decurrens*, as well as of all ptychodontid sharks. The resulting updated taxonomy, together with the description and reconstruction of the new crushing plate, greatly contribute to a better understanding of one of the most enigmatic families (Ptychodontidae) of Mesozoic elasmobranchs. The detailed investigation of the new dentition of *Ptychodus* from Dalmatia is also a further step towards the discovery of Upper Cretaceous ichthyofaunas of one of the most palaeontologically important areas of the Balkan Peninsula.

**Supplementary Information:**

The online version contains supplementary material available at 10.1186/s13358-024-00340-7.

## Introduction

The Dalmatian region (southern Croatia) is of great palaeontological interest, with well-preserved vertebrates such as fossil marine reptiles and fishes being repeatedly discovered in this area (Mekarski et al., 2019; Radovčić et al., 1983; Romer, 1966). In particular, Upper Cretaceous fish-bearing strata (“Ichthyoferous shales”) represented by platy limestones are well known from various hinterland (e.g., Trogir) and coastal (e.g., Hvar Island) areas of Croatia (Diedrich et al., 2011; Gorjanović-Kramberger, 1895; Radovčić et al., 1983; Stache, 1899). Numerous fossil fishes have been recovered especially from limestones at Primošten, Labišnica, and Prapatnica (Dalmatia, Croatia) as fragments, isolated specimens and, more rarely, complete skeletons (“Konzentrat–Lagerstätten”; Murray et al., 2016; Radovčić et al., 1983). From these localities, a very diverse ichthyofauna, including both elasmobranchs (e.g., Ptychodontidae and Carchariidae) and teleosts (e.g., Armigatidae, Tselfatiidae, Polymixiidae, Trachichthyidae, Holocentridae, Decertidae, Enchodontidae and Eurypholidae) were discovered (Japundžić et al., 2023; Murray et al., 2016; Radovčić et al., 1983). Although numerous valuable finds were recovered, only a small part of the fish fauna from the Dalmatian region has been described in detail so far. The remaining fossil material is therefore in urgent need of careful examination in order to identify the recovered specimens and properly assess the taxonomic composition and the faunal structure of this important Upper Cretaceous marine assemblage.

Identifying and classifying organisms (taxonomy) is undeniably the basis of most, if not all, theoretical and applied biological sciences, because the species (“morphospecies” in palaeontology) is a fundamental unit of biology (Guerra-García et al., 2008; Hughes & Labandeira, 1995; Schalk & Heijman, 1997). Consequently, proper taxonomic identification of species provides the primary backbone for a number of scientific disciplines of major topical and contemporary relevance (e.g., biodiversity and conservation; Guerra-García et al., 2008; Schalk & Heijman, 1997; White et al., 2022). In addition, taxonomic information can be incorporated into phylogenetic frameworks, if the available taxonomy of the study group is rigorously and continuously updated (Soul & Friedman, 2015). Unfortunately, the number of specialists and taxonomic studies is now decreasing and species identifications, documentations and records are often confusing, ambiguous, outdated and, sadly, overlocked (Gippoliti, 2020; Guerra-García et al., 2008; Raposo et al., 2021; Schalk & Heijman, 1997; Soul & Friedman, 2015; Wu et al., 2022). Although species identification is also a key aspect in a palaeontological context, most fossil taxa are based on poor diagnostic characters, limited numbers of specimens and fragmentary material (Hughes & Labandeira, 1995; Soul & Friedman, 2015). Particularly the study of new specimens requires a re-evaluation of original species classifications for a more rigorous interpretation of possible morphological variations and patterns due to the additional available material (Hughes & Labandeira, 1995). Despite great progress in recent decades, the taxonomy and systematics of most Mesozoic sharks is still in need of revision (e.g., Amadori et al., 2019b, 2020a, 2022b, 2023; Cappetta, 2012; Everhart, 2017; Hamm, 2020; Jambura et al., 2023, 2024; Shimada, 2012; Stumpf et al., 2023; Villalobos-Segura et al., 2023). The taxonomy and systematics of notorious enigmatic sharks, such as the large shell-crusher shark *Ptychodus* Agassiz, 1834, were the centre of intense debates for centuries (Amadori et al., 2020a; Bassani, 1886; Brignon, 2019; Cappetta, 2012; Casier, 1953; Cuny, 2008; Hamm, 2020; Hoffman et al., 2016; Nicholls, 2010; Parkinson, 1811; Patterson, 1966; Shimada, 2012; Vullo et al., 2024; Woodward, 1912).

*Ptychodus* (Ptychodontidae; Lamniformes) is an extinct elasmobranch mostly known from the Upper Cretaceous of North America, Europe, Africa, and Asia (e.g., Amadori et al., 2022b; Cappetta, 2012; Hamm, 2020; Vullo et al., 2024; Woodward, 1912). This predatory shark diversified greatly during the Late Cretaceous and reached its diversity peak during the Turonian-Coniacian (Amadori, 2022; Hamm, 2020). Isolated teeth, together with rare dental plates and mineralised cartilaginous elements, primarily make up the fossil record of this fossil shark (e.g., Amadori et al., 2019a, b, 2020a, b, 2022a; Everhart & Caggiano, 2004; Hamm, 2020; MacLeod, 1982; Shimada et al., 2009, 2010; Woodward, 1887, 1904, 1912). Within the genus *Ptychodus*, various cuspidate and un-cuspidate species can be identified based on general dental features together with occlusal ornamentations, such as ridges, granules and wrinkles (Amadori et al., 2020a, 2022a, 2023; Cappetta, 2012; Hamm, 2020; Shimada, 2012; Woodward, 1912). In *Ptychodus*, molariform teeth suitable to crush and/or grind shelled prey (durophagy) were arranged in massive tooth plates placed on the anteriormost part of V-shaped jaws (Amadori et al., 2020a; Hamm, 2020; Shimada et al., 2009; Woodward, 1904, 1912). Species of *Ptychodus* characterised by different tooth morphologies could have targeted different main prey while still keeping a varied diet (e.g., Amadori et al., 2020b; Shimada, 2012). In general, their diet range could have spanned from hard-shelled prey (e.g., bivalves, ammonites and turtles) to soft-bodied prey (e.g., Amadori et al., 2019a, 2020b, 2023; Cappetta, 2012; Everhart, 2017; Hamm, 2020; Shimada, 2012; Vullo et al., 2024). Recently, complete skeletons of *Ptychodus* have been documented for the first time from the Turonian (Upper Cretaceous) of Mexico revealing its close relationship with extinct and extant lamniform sharks (Vullo et al., 2024).

Among the un-cuspidate species, *Ptychodus decurrens* Agassiz, 1838 is well known for having molariform teeth with thin, branched ornamentations (e.g., Cappetta, 2012; Woodward, 1912). As for the other species, most of the fossil record of *P. decurrens* consists of isolated teeth (Cappetta, 2012). *Ptychodus decurrens* has been primarily reported from upper Cenomanian–middle Turonian sedimentary rocks with a palaeogeographical distribution covering numerous localities around the world (e.g., Alvarado-Ortega et al., 2006; Amadori et al., 2022b, 2023; Amalfitano et al., 2020; Antunes & Cappetta, 2002; Cappetta, 2012; Carrillo-Briceño, 2009; Hamm, 2020; Hoch, 1992; Radwański & Marcinowski, 1996; Siverson, 1999; Underwood & Cumbaa, 2010; Verma et al., 2012; Vullo & Courville, 2014). Few articulated tooth sets mostly preserving the centre of the upper and / or lower dentitions, and rare cartilaginous elements (jaws and vertebral centra), are only known from England, Germany and USA (see Müller, 2008; Williamson et al., 1991; Woodward, 1887, 1904, 1912). Among the well-preserved skeletons of *Ptychodus* documented from the Upper Cretaceous of Mexico, one specimen shows associated teeth clearly having the typical occlusal ornamentation of *P. decurrens* (see Vullo et al., 2024: figs. 2d, S9c).

Here, a new articulated tooth plate of *Ptychodus decurrens* Agassiz, 1838 from the Turonian (Upper Cretaceous) of Dalmatia (southern Croatia) is described. This nearly complete dentition allows the reconstruction of the entire lower crushing plate of *P. decurrens*. Moreover, the un-cuspidate species *P. decurrens* is reviewed based on the careful examination and redescription of the original types and relevant comparative material. Taxonomic and nomenclatural issues related to *P. decurrens* are thus addressed here. Species-specific characters of *P. decurrens* are discussed and compared here with those characterizing the morphologically closest taxa to clarify the taxonomic ambiguity accumulated over the years. Lectotype and paralectotypes of *P. decurrens* are selected here in order to ensure stability of the taxonomic status of the species. Moreover, the redescribed specimens previously attributed to various dubious taxa are reassigned to *P. decurrens*.

## Historical background

**Fig. 1:**
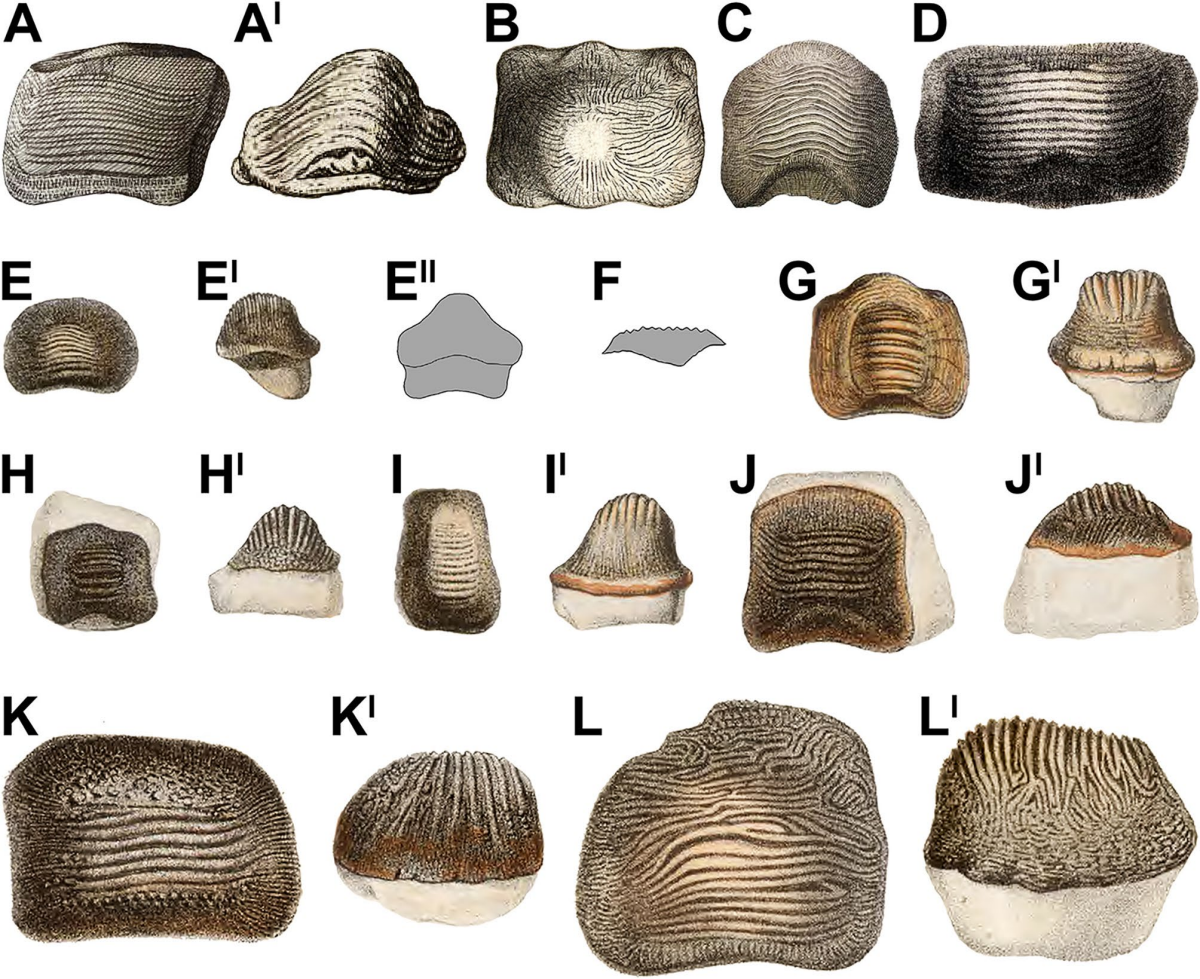
Historical images of teeth of *Ptychodus* Agassiz, 1834 from the Upper Cretaceous of Europe. The specimens are in occlusal (**A, B–E, G, H, I, J, K, L**), posterior (**A**^**I**^**, E**^**II**^) and lateral (**E**^**I**^**, F, G**^**I**^**, H**^**I**^**, I**^**I**^**, J**^**I**^**, K**^**I**^**, L**^**I**^) views. **A–D** isolated teeth of *P. decurrens* Agassiz, 1838. **A, A**^**I**^ isolated tooth originally figured by Brückmann (1752: pl. 5, fig. 4, pl. 6, fig. 4). **B** Séguier’s unpublished isolated tooth (courtesy of A. Brignon); **C** isolated tooth originally figured by Parkinson (1811: pl.18, fig. 12); **D** isolated tooth originally figured by Passy (1832: pl. 15, fig. 4); **E–L**^**I**^ type series originally figured and identified by Agassiz (1838: pl. 25b, figs. 1–8) as to *P. decurrens* and here reassigned to different species of *Ptychodus* (**E–E**^**II**^ isolated tooth of *P. decurrens*; **F** isolated tooth fragment of *Ptychodus* sp.; **G–H**^**I**^ isolated teeth of *P. mammillaris* Agassiz, 1835; **I–I**^**I**^ isolated tooth of *P. altior* Agassiz, 1835; **J–L**^**I**^ isolated teeth of *P. decurrens*). Silhouettes in grey (**E**^**II**^**, F**) are based on original drawings by Agassiz (1838: pl. 25b, figs. 1^II^, 2)

Isolated teeth of the extinct shark *Ptychodus decurrens* Agassiz, 1838 have been known for more than two centuries and were among the first to be documented and figured within the genus (Brignon, 2019). However, those were commonly misidentified as dental remains of porcupinefishes (Diodontidae; Actinopterygii) due to the morphological similarity between these isolated finds and the fossil palates of the genus *Diodon* Linnaeus, 1758 (see also Amadori et al., 2019a; Brignon, 2019). Among the first teeth of *Ptychodus* Agassiz, 1834 that ever were published, Brückmann (1752: pl. 5, fig. 4, pl. 6, fig. 4) figured one tooth of *P. decurrens* (“Harrer” collection; see Fig. 1A) from the upper Cenomanian of Kneiting (Bavaria, south-eastern Germany; Brignon, 2019). Between 1740 and 1750, Jean-François Séguier worked on a manuscript entitled “Pétrifications du Veronois”, which included plates depicting fossil remains from the Upper Cretaceous of the Verona province (northern Italy; Brignon, 2019: p. 13–15, fig. 8). Séguier’s incomplete study remained unpublished until the XXI century (Gaudant, 2005; see also Brignon, 2019). One of the teeth depicted by Séguier shows very similar, if not identical, ornamentation patterns to those of *P. decurrens* (see Fig. 1B; see also Brignon, 2019). Despite a slight resemblance also to the ornamentation of *P. occidentalis*, the large raised occlusal area suggests a greater affinity with *P. decurrens*. It is interesting to note that the occlusal surfaces of the teeth figured by Brückmann and Séguier are convex rather than being flat (see Fig. 1A, B). Later, Parkinson (1811: pl.18, fig. 12; see Fig. 1C) and Passy (1832: pl. 15, fig. 4; see Fig. 1D) figured two teeth of *P. decurrens* from the Upper Cretaceous of southern England and northern France, respectively, but originally identifying them as parts of palates of unknown fish (see also Brignon, 2019: p. 26, 47, fig. 18C, 32N).

**Fig. 2:**
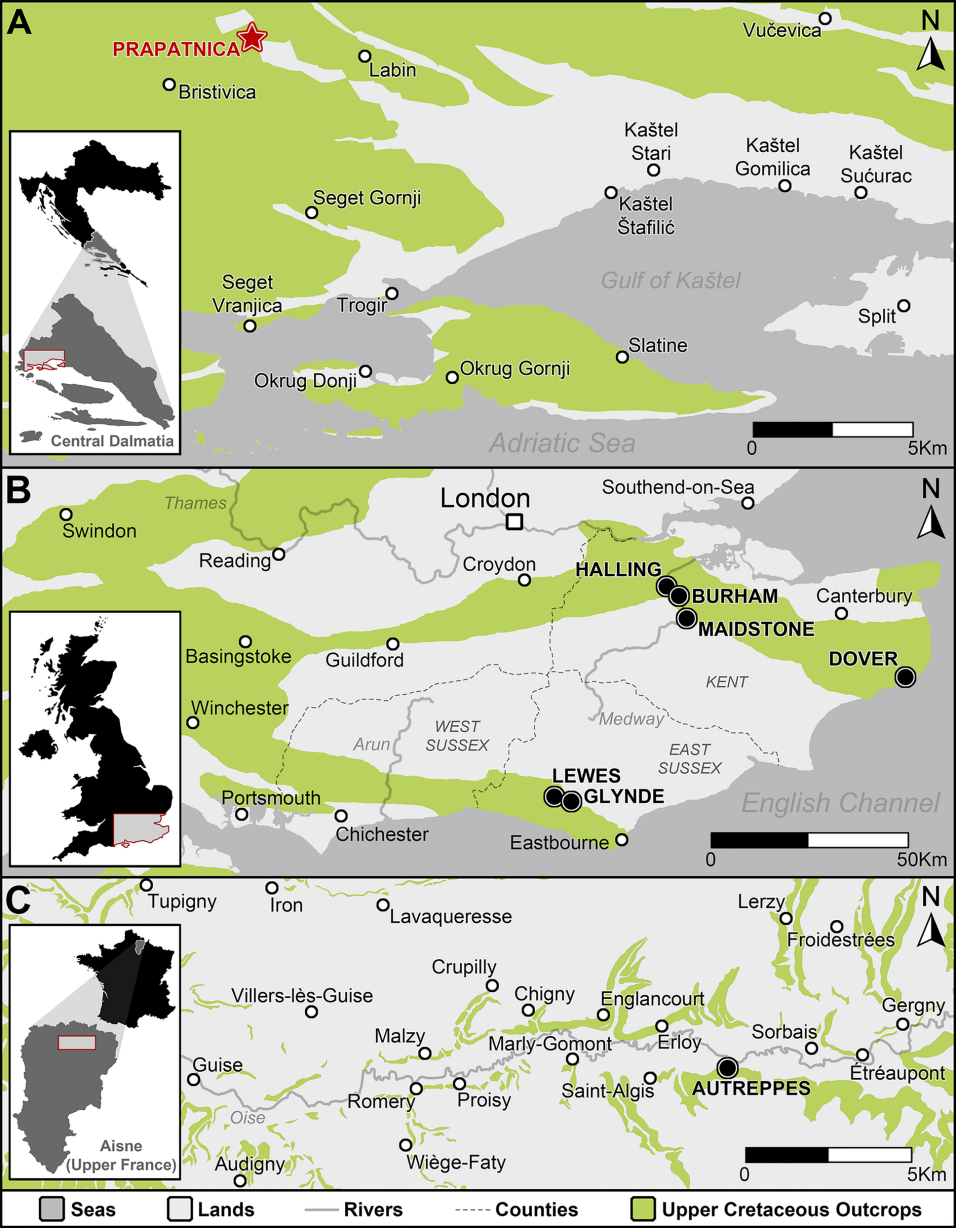
Location map showing the sites of provenance of the study material. **A** locality of provenance of the new articulated dentition (see red star) from southern Croatia (based on Marinčić et al., 1971); **B** localities of provenance of the comparative material (see black dots) from south-eastern England (based on Friedmann et al., 2016); **C** localities of provenance of the comparative material (see black dots) from northern France (based on Celet et al., 1979 and Vernhet, 2008)

**Fig. 3:**
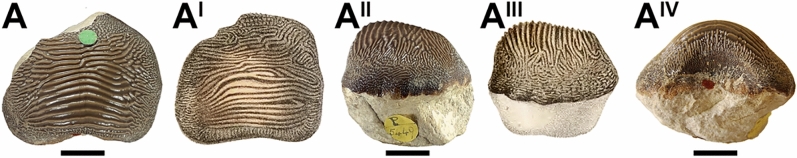
Isolated tooth (NHMUK PV OR 5449) designated herein as lectotype of *Ptychodus decurrens* Agassiz, 1838. **A, A**^**I**^ occlusal view; **A**^**II**^**, A**^**III**^ lateral view; **A**^**IV**^ posterior view. **A**^**I**^**, A**^**III**^ images from Agassiz (1838: pl. 25b, figs. 8, 8I). All scale bars equal 10 mm

**Fig. 4:**
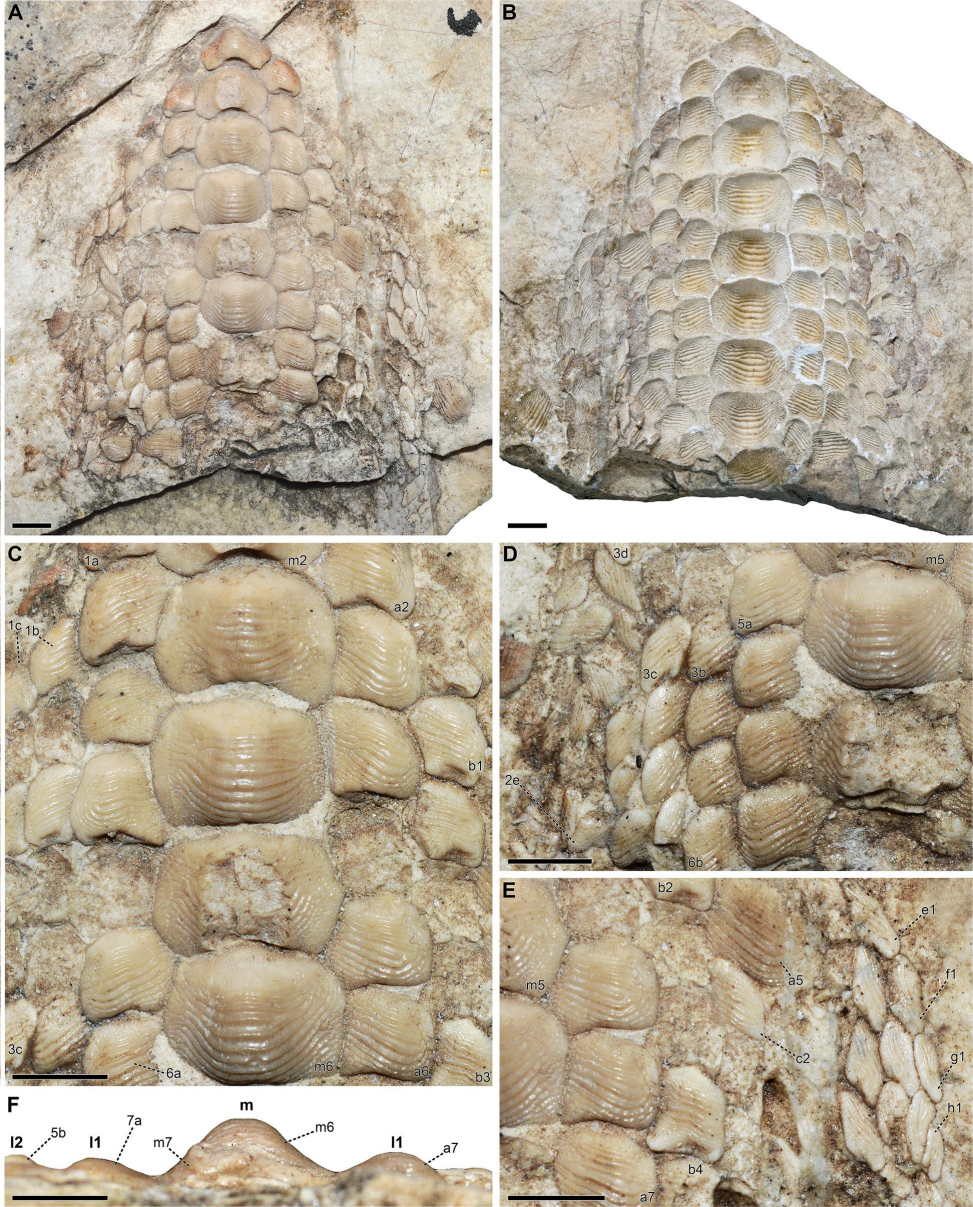
Lower tooth plate (CNHM 9350) of *Ptychodus decurrens* Agassiz, 1838 from the Turonian of Croatia. The articulated specimen is figured in occlusal (**A–E**) and posterior (**F**) views. **A, B** part and counterpart of the whole specimen; **C–F** details of the articulated teeth. For the tooth codes (e.g., m2, a2, etc.) see the “Interpretative drawing” in Fig. 5. All scale bars equal 5 mm

**Fig. 5:**
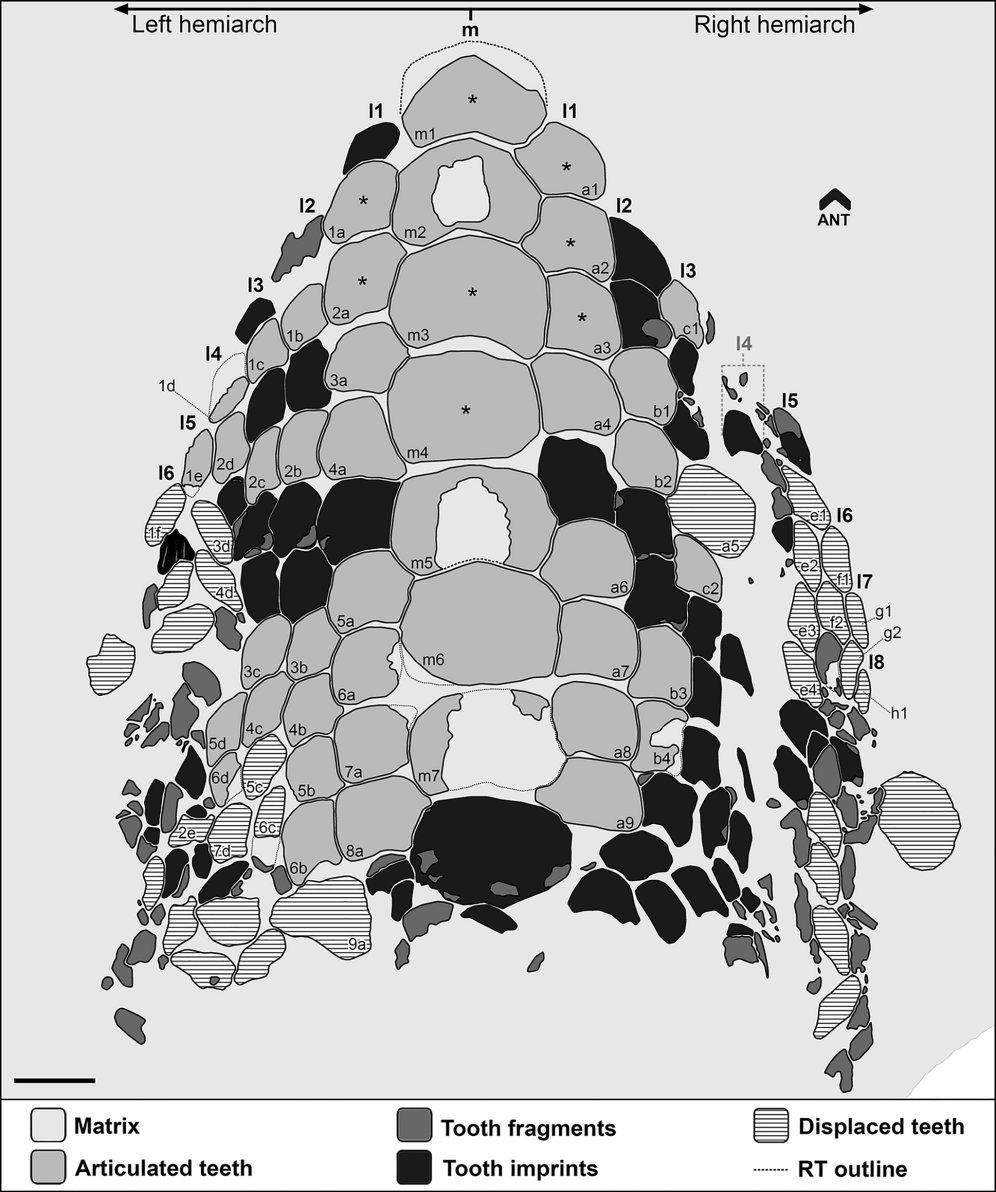
Interpretative line drawing of the lower dentition (CNHM 9350) of *Ptychodus decurrens* Agassiz, 1838. Asterisks indicate the worn teeth. A code (e.g., m1, a1, a2, etc.) has been arbitrarily assigned to all the teeth preserved from anterior end of each tooth row. m, symphyseal row; l1–8, lateral rows. RT, reconstructed teeth. Scale bar equals 5 mm

**Fig. 6:**
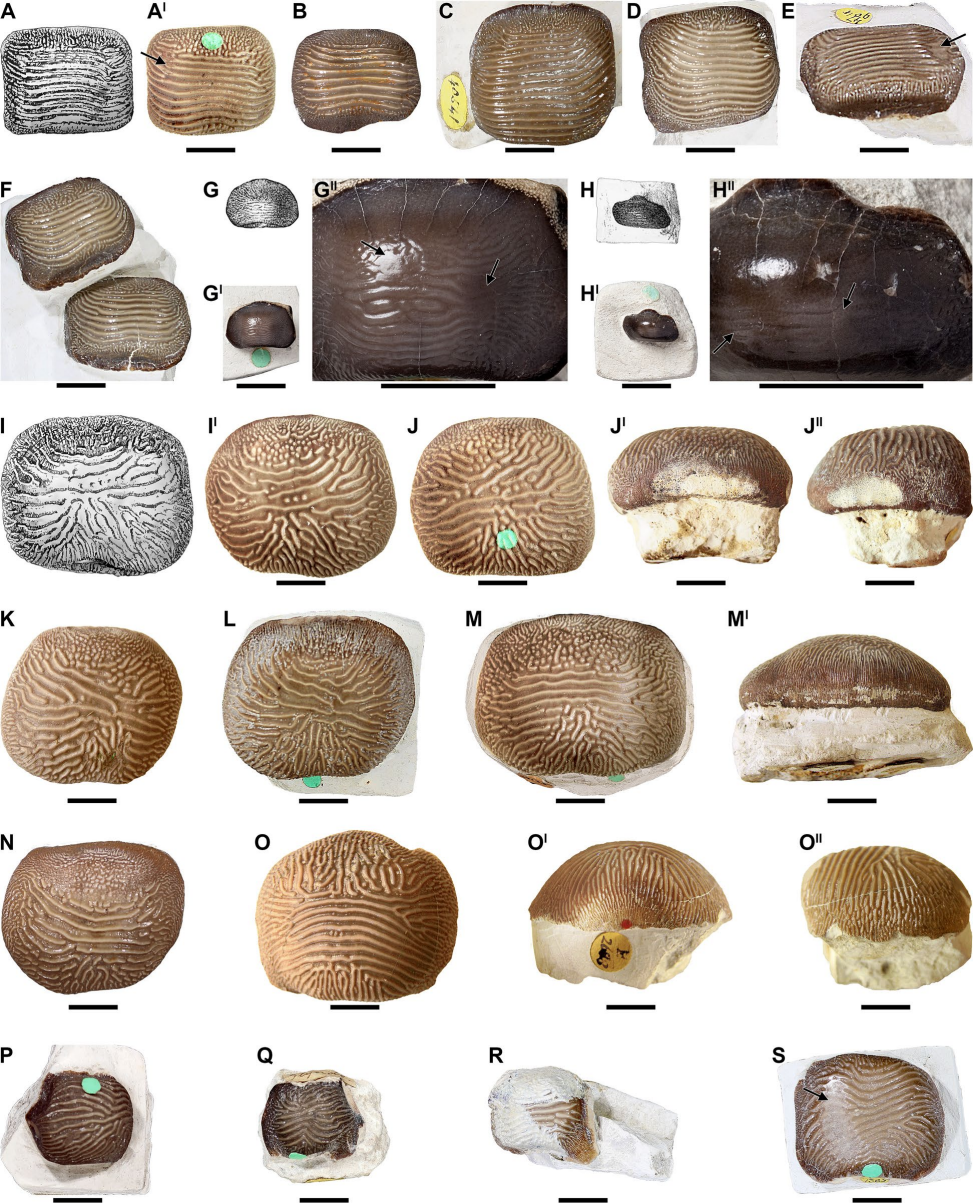
Disarticulated teeth from the Upper Cretaceous of England assigned here to *Ptychodus decurrens* Agassiz, 1838. The specimens are figured in occlusal (**A–J, K–M, N, O, Q–S**), anterior (**J**^**I**^**, M**^**I**^), posterior (**O**^**I**^) and lateral (**J**^**II**^**, O**^**II**^) views. **A, A**^**I**^ isolated tooth NHMUK PV OR 28348; **B** upper lateral tooth NHMUK PV OR 28349; **C** isolated tooth NHMUK PV OR 40541; **D** associated tooth NHMUK PV OR 47904a; **E** associated tooth NHMUK PV OR 47904b; **F** associated teeth NHMUK PV OR 47904c; **G–G**^**II**^ isolated tooth NHMUK PV P 6524; **H–H**^**II**^ isolated tooth NHMUK PV P 51; **I, I**^**I**^ associated tooth NHMUK PV OR 39125a; **J–J**^**II**^ associated tooth NHMUK PV OR 39125b; **K** associated tooth NHMUK PV OR 39125c; **L** associated tooth NHMUK PV OR 39125d; **M–M**^**I**^ associated tooth NHMUK PV OR 39125e; **N** isolated tooth NHMUK PV OR 28342; **O–O**^**II**^ isolated tooth NHMUK PV P 2688; **P** isolated tooth NHMUK PV 9718; **Q** isolated tooth NHMUK PV OR 49855; **R** isolated tooth NHMUK PV 9029a; **S** isolated tooth NHMUK PV P 1385. Arrows indicates occlusal tooth wear. Scale bars equal 10 mm (**A–G**^**I**^**, H, H**^**I**^**, I–S**) and 5 mm (**G**^**II**^**, H**^**II**^)

**Fig. 7:**
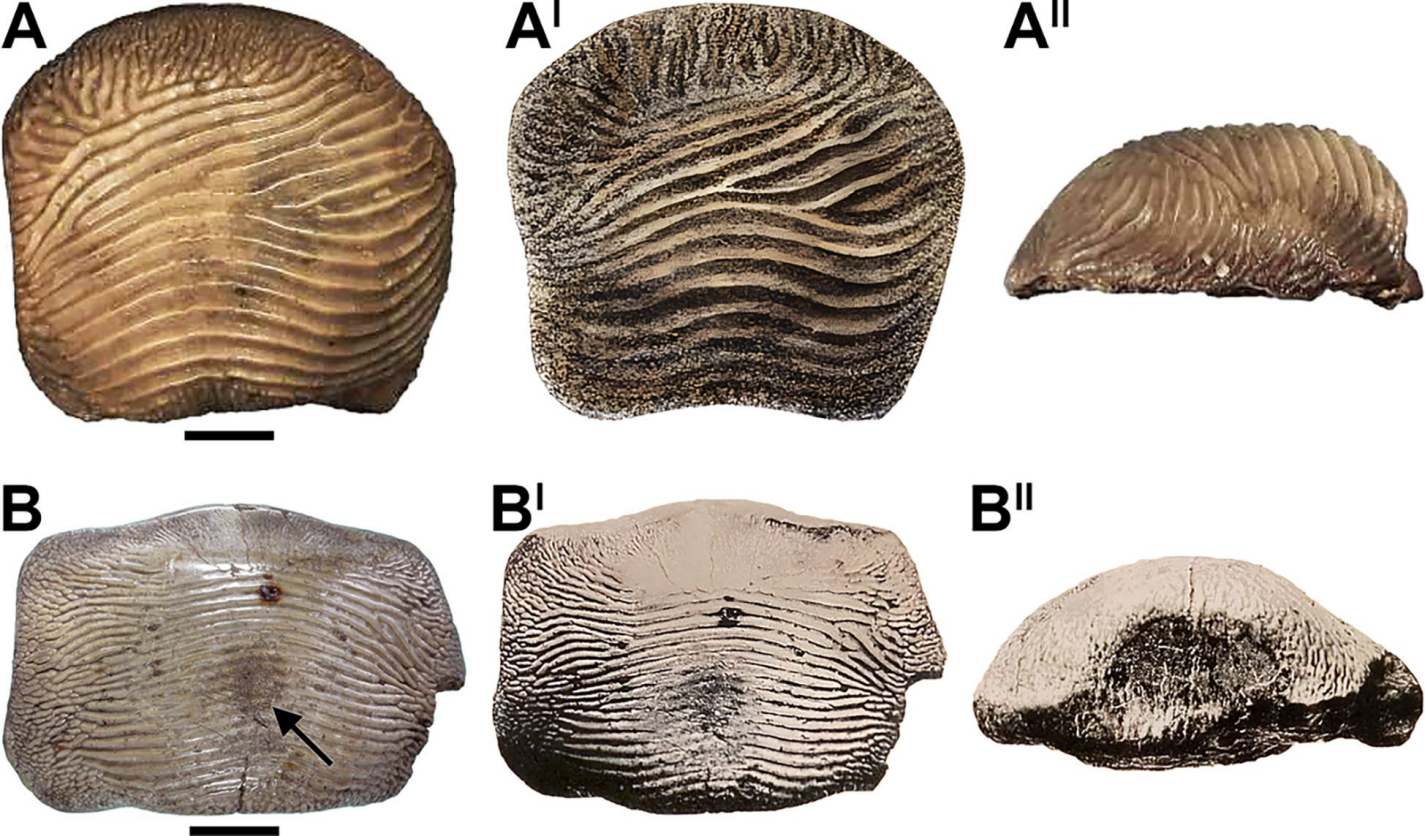
Isolated teeth reassigned here to *Ptychodus decurrens* Agassiz, 1838. The specimens come from the Upper Cretaceous of England (**A–A**^**II**^) and northern France (**B–B**^**II**^) and are figured in occlusal (**A, A**^**I**^**, B, B**^**I**^), posterior (**B**^**II**^) and lateral (**A**^**II**^) views. **A–A**^**II**^ lateral tooth MNHN.F.CTE221; **B–B**^**II**^ symphyseal tooth MGL 6263. Arrows indicates occlusal tooth wear. All scale bars equal 10 mm

**Fig. 8:**
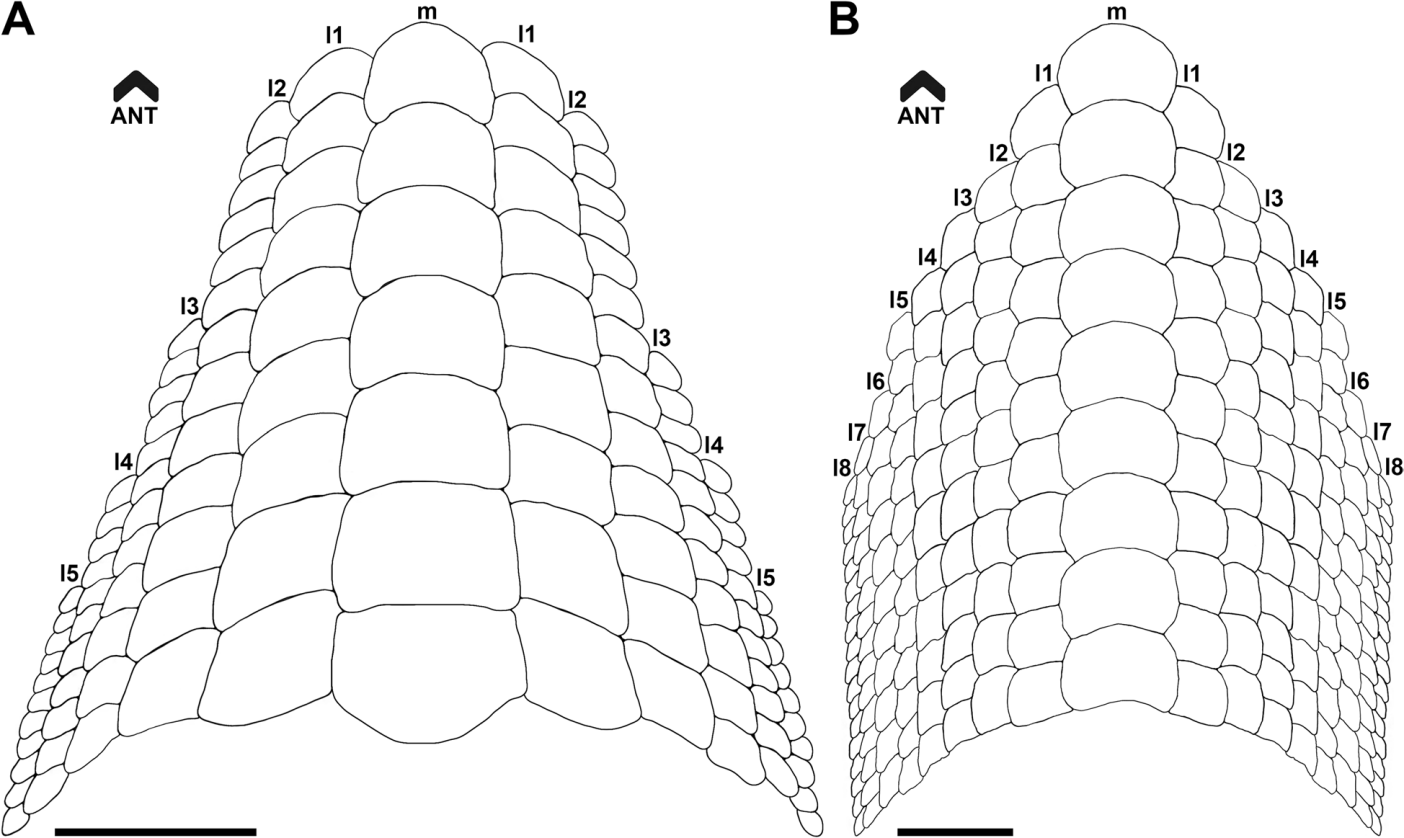
Interpretative reconstructions of the lower tooth plates of *Ptychodus decurrens* Agassiz, 1838. **A** reconstruction modified after Woodward (1904: text fig. on p. 134); **B** reconstruction based on the new articulated dentition (CNHM 9350). The replacement teeth are not represented in both interpretative drawings. All scale bars equal 10 mm

Agassiz (1835) mentioned for the first time the species *Ptychodus decurrens* in his “Feuilleton additionnel n. 54” of the “Recherches sur les poissons fossiles”, but without referring to any type specimen for this new name (see Brignon, 2015, 2019). According to art. 12 of the International Code of Zoological Nomenclature (ICZN, 1999), publications intended to erect new taxa before 1931 have to include a description or an illustration, or clearly refer to a previously published description or illustration, of one or more specimens (type material; see also art. 12.2.7). Moreover, those have to provide the objective standard of reference (unambiguous morphological characters) for the application of the name (see “Principle of Typification” in art. 61 of ICNZ, 1999). Failing to conform to art. 12 of the ICZN (1999), *P. decurrens* Agassiz, 1835 has to be simply regarded as “*nomen nudum*” (see also Brignon, 2015, 2019). Later, Agassiz (1838) associated the scientific name *P. decurrens* to eight isolated teeth (see Fig. 1E–L) from southern England on plate 25b, figs. 1–8 of his “Recherches sur les poissons fossiles”, establishing the taxon officially (see ICZN, 1999: art. 12; see also Brignon, 2015, 2019). Subsequently, a first description of the species was also provided by Agassiz (1839: 154–155). Since then, numerous isolated, and associated specimens of *P. decurrens* from almost all over the world have been described up to now (e.g., Amadori et al., 2022b, 2023; Antunes & Cappetta, 2002; Carrillo-Briceño, 2009; Herman, 1977; Siverson, 1999; Underwood & Cumbaa, 2010; Verma et al., 2012; Williamson et al., 1991). In addition, Woodward (1887: p. 23, pl. 10, figs. 1–10; see also Woodward, 1912: text fig. 70, 76, 77) described and figured for the first time articulated tooth sets of *P. decurrens* Agassiz, 1838 from the Upper Cretaceous of Sussex and Kent (southern England) and, later, provided the first reconstruction of a complete lower dental plate of this un-cuspidate species based on a few articulated teeth (see Woodward, 1904: text fig. on p. 134).

## Geological setting

The new tooth plate (CNHM 9350) described here was found in the surrounding of the village of Prapatnica (Dalmatia, southern Croatia; see Fig. 2A). The exact location is close to the “Bojić” houses, on the northern slope of Vilaja hill. This area is located 10 km NNE and 15 km NNW, respectively, of Trogir city. The tooth plate is preserved in wackestone/packstone limestones with oligosteginids and calcitised radiolarian at very low frequency. The limestones in Prapatnica exhibit sporadic turbidite layers and are indicative of inner carbonate platform environment, possibly the inner slope with significant communication with an off-reef area (Radovčić et al., 1983). The age of the outcrops is traditionally considered to be of “Senonian” (possibly Campanian; et al., 2016; Japundžić et al., 2023; Murray et al., 2016; Radovčić et al., 1983). The deposition took place on Adriatic carbonate platform, one of the largest Mesozoic carbonate platforms of the peri-Tethyan seas (Vlahović, 2005). The Adriatic platform was characterised mostly by shallow-marine deposition, with numerous short or long periods of emergence, as a consequence of the interaction of synsedimentary tectonics and eustatic changes (Vlahović, 2005). Several fossil fishes were found from the Prapatnica locality in 1972, including three holotypes already described (Murray et al., 2016; Radovčić, 1975).

The type specimens of *Ptychodus decurrens* Agassiz, 1838 and the comparative material mostly come from various localities of the Upper Cretaceous Chalk of southern England, more precisely from Kent and East Sussex and surrounding counties (see Fig. 2B). The remains are referred to the classic “Lower Chalk Group” (Amalfitano et al., 2022; Hopson, 2005), which is part of the Chalk Group, specifically the Grey Chalk Subgroup. The sediments of the subgroup were deposited in the north-western part of the Anglo-Paris Basin and is equivalent to the interval including, from bottom to top, the West Melbury Marly Chalk Formation (with the Glauconitic Marl Member at the bottom), the Zig Zag Chalk Formation and the Plenus Marls Member of the Holywell Nodular Chalk Formation in the Southern Province (Gale, 2019; Hopson, 2005; Wilkinson, 2011). The “Lower Chalk” spans from the early Cenomanian to late Cenomanian (Hopson, 2005; Wilkinson, 2011) and yielded remains of numerous fossil fish taxa, although the number of vertebrates per volume of rock is very low (Amalfitano et al., 2022; Friedman et al., 2016; Kriwet, 2002; Mantell, 1822; Woodward, 1902, 1903, 1907, 1908, 1909, 1911, 1912). The fish remains include numerous bony fishes and chondrichthyans (e.g., Dixon, 1850; Kriwet, 2002; Leriche, 1902; Longbottom & Patterson, 2002; Woodward, 1902, 1903, 1907, 1908, 1909, 1911, 1912).

A single isolated tooth (MGL 6263) used here for comparison comes from the Upper Cretaceous of “Autreppe” (now Autreppes) surrounding (near Guise, Aisne, Upper France; see Fig. 2C). Outcrops in the Guise area span from early Cenomanian to late Coniacian and are described by Celet et al. (1979). The succession includes glauconitic sandstones, clays and marls within the interval corresponding to the Cenomanian, while the lower Turonian and overlying interval up to the Coniacian is represented by calcareous clays and clayey marls, followed at the top by marly chalk (middle Turonian), white and grey chalk (upper Turonian) and grey beige to white chalk (Coniacian). Outcrops in the surrounding of Autreppes are mainly upper Cenomanian grey-white, chalky limestones often masked by ancient alluvium, flinty surface formations or even solifluous Turonian marls. The palaeoichthyological content of the Cenomanian of Autreppes is poorly known with a few exceptions. For instance, Leriche (1902) figured teeth of sharks (*Scapanorhynchus*, *Cretoxyrhina*) and mentioned coprolites of coelacanths (*Macropoma*).

## Materials and methods

### Materials

The new articulated tooth plate CNHM 9350 from Dalmatia (southern Croatia) presented here is housed at the Croatian Natural History Museum in Zagreb. The dentition was originally collected during field work in 1972 as part of a joint project on Cretaceous teleostean fishes of Yugoslavia. The project was led by Professor David Bardack (University of Illinois, Chicago) in cooperation with Ivan Crnolatac (Geological and Paleontological Museum, Zagreb) and Jakov Radovčić (Institute for Paleontology and Quaternary Geology of the Yugoslav Academy of Sciences and Arts, Zagreb). For more information on the field work see Bardack and Radovčić (1973) and Radovčić (1975).

In addition, two tooth sets (catalogue numbers: NHMUK PV OR 47904 and NHMUK PV OR 39125) and fourteen isolated teeth (catalogue numbers: MNHN.F.CTE221, MHNN FOS. 474, NHMUK PV OR 28342, NHMUK PV OR 28348, NHMUK PV OR 28349, NHMUK PV OR 40541, NHMUK PV OR 49855, NHMUK PV P 51, NHMUK PV P 1385, NHMUK PV P 2688, NHMUK PV OR 5449, NHMUK PV P 6524, NHMUK PV P 9029a and NHMUK PV P 9718) from England and an isolated tooth (MGL 6263) from France were selected and re-described here for comparative purposes in order to provide a detailed revision of the species *Ptychodus decurrens* Agassiz, 1838. See Table S1 in “Additional file 1” (supplementary material) for details of the specimens.

Additional comparative material examined here includes three articulated dentitions of *P. decurrens* (BMB 008524, BMB 008605a-g and NHMUK PV P 10336), the type series of *P. multistriatus* (NHMUK PV P 2681) and the type series of *P. occidentalis* (ANSP 1460–1466). The redescription and detailed investigation of these additional specimens are the focus of other studies currently in progress (e.g., Amadori et al. in prep).

### Methods

A Nikon D3200 camera with a mounting 18–55 mm Nikon macro lens was used to create high-quality photos of the dentition from Croatia and the tooth from France, while a Nikon D810 camera with a mounting 60–90 mm lens and a Canon PowerShot SX720 HS were used to take pictures of the material from England. The illustrative drawings and images of the fossils were prepared using the software package Photoshop CS6 (v.25.0). The method employed to reconstruct the lower dental plate of *Ptychodus decurrens* Agassiz, 1838 follows Amadori et al. (2020a). A smear slide of the matrix embedding the new specimen CNHM 9350 was prepared for calcareous nannofossil analysis to date the specimen (for more details, see Amadori et al., 2020a). The calcareous nannofossil analysis was performed on an area of 6–7 mm^2^ (roughly 3 vertical traverses; modified after Gardin & Monechi, 1998). Anatomical and odontological terminologies used here follow Cappetta (2012) with a few exceptions. Each linguo-labial (or antero-posterior) succession of teeth is here indicate as a “tooth row”, as proposed by Shimada (2002: p. 42); see also Compagno, 1988; Welton & Farish, 1993). Although in disagreement with the mesio-distal alignment of the tooth rows adopted by Cappetta (2012: p. 10), the definition offered by Shimada (2002) best fits the arrangement and orientation of the teeth within the crushing plates of *Ptychodus* Agassiz, 1834. Open nomenclature follows the standards proposed by Matthews (1973), Bengtson (1988) and Sigovini et al. (2016).

### Institutional abbreviations

**ANSP**, The Academy of Natural Sciences of Drexel University, Philadelphia, USA; **BMB**, Booth Museum of Natural History, Brighton, UK; **CNHM**, Croatian Natural History Museum, Zagreb, Croatia; **MGL**, Lille Natural History Museum, Lille, France; **MHNN**, Muséum d’Histoire naturelle de Neuchâtel, Neuchâtel, Switzerland; **MNHN**, National Museum of Natural History, Paris, France; **NHMUK**, Natural History Museum, London, UK; **RSM**, Royal Saskatchewan Museum, Regina (Saskatchewan), Canada.

## Results

### Systematic palaeontology

Class **Chondrichthyes** Huxley, 1880

Subclass **Elasmobranchii** Bonaparte, 1838

Order **Lamniformes** Berg, 1937

Family **Ptychodontidae** Jaekel, 1898

Genus ***Ptychodus*** Agassiz, 1834

***Type species. Ptychodus schlotheimii*** Agassiz, 1834 (*nomen oblitum*), senior synonym of *Ptychodus latissimus* Agassiz, 1835 (*nomen protectum*). See Giusberti et al. (2018).

***Diagnosis.*** See Vullo et al., (2024: p. 3).

***Ptychodus decurrens*** Agassiz, 1838

(Figs. 1A–E^II^, J–L^I^, 3–8).

Selected synonyms list (for the complete list, see “Additional file 2” in supplementary material):

p.1752 *Dentem seu palatum piscis Ostracionis*; Brückmann: p. 116; pl. 5, fig. 4 (non fig. 4).

p.1752 “ipsum dentem exemtum petrefactum”; Brückmann: p. 120; pl. 6, fig. 4.

*1835 Ptychodus decurrens* Ag.; Agassiz: p. 54 (*nomen nudum*).

*vp.1838 *Ptychodus decurrens* Agass.; Agassiz: atlas vol. 3, pl. 25b, figs. 1, 68 (non figs. 3–5).

vp.1838 *Ptychodus polygyrus* Agass.; Agassiz: atlas vol. 3, pl. 25b, fig. 21 (non figs. 4-8, 10, 11).

1839 *Ptychodus decurrens* Agass.; Agassiz: vol. 3; p. 154.

vp.1839 *Ptychodus polygyrus* Agass.; Agassiz: atlas vol. 3, pl. 25, fig. 9 (non figs. 4–8, 10, 11).

v1850 *Ptychodus decurrens*; Dixon: p. 362; pl. 30, fig. 7, 8; pl. 31, fig. 1; pl. 32, fig. 5.

v1850 *Ptychodus depressus* new; Dixon: p. 363; pl. 31, fig. 9.

v1850 *Ptychodus Oweni* new; Dixon: p. 364; pl. 31, fig. 2.

1887 *P. decurrens*; Woodward: p. 123; pl. 10, figs. 2–10, 13.

1893 *Ptychodus levis,* sp. nov.; Woodward: p. 192; pl. 5, figs. 5, 6.

1902 *Ptychodus decurrens* var. *multiplicatus* nov. var.; Leriche: p. 96; pl. 2, fig. 20.

v1904 *Ptychodus decurrens*; Woodward: p. 133; text fig., p. 134; pl. 15, figs. 1–5.

v1912 *Ptychodus decurrens* Agassiz; Woodward: p. 239; text fig. 70 on p. 226; text fig. 71 on p. 227; text fig. 76 on p. 241, text fig. 77 on p. 243; pl. 51, pl. 52 (non syn.).

1973 *Ptychodus decurrens* Agassiz L. 1835 var. decurrens nov. var.; Herman: atlas, pl. 2, fig. 1.

1977 *Ptychodus decurrens* Agassiz L. 1835; Herman: p. 49; text fig., p. 52 (non syn.).

1977 *Ptychodus oweni* Dixon F. 1850; Herman: p. 53; text fig., p. 53.

2008 *Ptychodus decurrens* Agassiz, 1835; Müller: p. 62; pl. 1, 2.

p.2016 *Ptychodus* sp.; Hoffman: p. 743; figs. 3.7–9, 14 (non figs. 3.1–6, 7–13).

2019 *Ptychodus decurrens* Agassiz, 1838; Brignon: p. 6, figs. 1, 5C.4, D.4, 8B.5, 8D, 18C, 44, 51C (middle figure), 52B–E, 54 (bottom figure, p. 70), 57 (three middle figures), 58A, B.

2020 *Ptychodus decurrens* Agassiz, 1835; Hamm: p. 26; figs. 37–48.

v2024 *P. decurrens*; Vullo et al.; p. 3, fig. 2d.

**Type series**. The eight isolated teeth from southern England illustrated by Agassiz (1838: pl. 25b, figs. 1–8; see Fig. 1E–L^I^) and assigned to *Ptychodus decurrens*. According to the ICZN (1999: art. 74), we designate herein the lectotype of *P. decurrens* Agassiz, 1838 in order to fix and clarify the taxonomic identity of the species (see below).

**Lectotype**. NHMUK PV OR 5449 (Fig. 3) is an isolated tooth from the “Mantell collection” housed in the Natural History Museum, London and it is designated herein as lectotype. The specimen corresponds to the tooth originally illustrated by Agassiz (1838) on plate 25b, figs. 8, 8' (Fig. 1L; see also Brignon, 2019: p. 70). NHMUK PV OR 5449 (Fig. 3) has a massive crown with symmetric outline, deep posterior sulcus and bulgy occlusal surface. The anterior protuberance is broken on the left side (see Fig. 3A). Fifteen thin ridges cross the tooth occlusal surface. The ends of the ridges reach the marginal zone where they taper off and branch out to the edges of the tooth crown (see Fig. 3A, A^II^). The ridges merge with thin wrinkles on the marginal areas composing a fine reticulation. The marginal ornamentation also includes rare granules mostly occurring on the anterior side (see Fig. 3A). The root of NHMUK PV OR 5449 is broken. In lateral view (Fig. 3A, A^II^), the outline of its crown is tilted anteriorly. In posterior view (Fig. 3A^IV^), the raised central area of the tooth crown has a large base and a rounded top.

**Paralectotypes**. The remaining seven specimens of the original type series figured by Agassiz (1838: pl. 25b, figs. 1–7; see Fig. 1E–K^I^) have to be considered as paralectotypes since they were originally syntypes of *Ptychodus decurrens* Agassiz, 1838, together with the selected lectotype NHMUK PV OR 5449 (Fig. 3; see ICZN, 1999; arts. 73.2.2, 74.1.3 and recommendation 74F; see also Brignon, 2019). However, three of these specimens (see Fig. 1G–I^I^) are clearly assignable to other species (*P. altior* Agassiz, 1835 and *P. mammillaris* Agassiz, 1835; see also Amadori et al., 2019b). Among the teeth originally figured by Agassiz (1838), those in Fig. 1E, J–L^I^ exhibit all the typical characters of *P. decurrens* (see “Diagnosis”, below) and, therefore, they have to be considered belonging to the species. The major morphological differences between them concern the general shape of their crown. Most of these specimens exhibit a transversally elongated dental crown (e.g., Fig. 1E, K). In contrast, MHNN FOS. 474 (Fig. 1J) is quite squared (see also Brignon, 2019: fig. 52D, E). Only one tooth (Fig. 1K) has a relatively flat occlusal surface (see Fig. 1K^I^), while the others show bulged crowns (see Fig. 1E^I^, E^II^, J^I^, L^I^).

**Emended Diagnosis.** A *Ptychodus* species with square to rectangular tooth crowns crossed by thin and transversally elongated ridges; the lateral ends of the ridges reach the occlusal marginal area where they taper off and branch out to the edge of the tooth crown. Occlusal ornamentations with lateral loops and posterior ramifications may occur, but they are rare. Fine wrinkles arranged in an irregular reticulation pattern cover the poorly developed marginal areas. Granules are mostly limited to the anterior occlusal surface. Dental crowns are bulged at the centre of the lower dentition, while the distal areas consist of flat teeth. Upper dentition with flat or slightly convex teeth. Lower symphyseal teeth are more symmetrical and transversely elongated than lateral teeth. Central tooth rows are juxtaposed with each other, while the more distal ones show slight imbrication. Tooth size, bilateral symmetry and number of ridges decreasing mesiodistally across the dentition with the only exception of the upper symphyseals that are small and transversely compressed. Distalmost teeth are transversally elongated and almost triangular in shape.

**Referred material**. The articulated dentition CNHM 9350 from the Upper Cretaceous of Dalmatia (southern Croatia) housed at the Croatian Natural History Museum in Zagreb. Two tooth sets (NHMUK PV OR 47904 and NHMUK PV OR 39125) and fourteen isolated teeth (MNHN.F.CTE221, MHNN FOS. 474, NHMUK PV OR 28342, NHMUK PV OR 28348, NHMUK PV OR 28349, NHMUK PV OR 40541, NHMUK PV OR 49855, NHMUK PV P 51, NHMUK PV P 1385, NHMUK PV P 2688, NHMUK PV P 5449, NHMUK PV P 6524, NHMUK PV P 9029a and NHMUK PV P 9718) from England and an isolated tooth (MGL 6263) from France. The tooth set NHMUK PV OR 39125 consist of five detached teeth labelled here “NHMUK PV OR 39125a–e”. The tooth set NHMUK PV OR 47904 consists of four teeth embedded in three matrix blocks labelled here “NHMUK PV OR 47904a–c”. See Table S1 in “Additional file 1” (supplementary material), for details of the specimens.

**Occurrences and ages.** The calcareous nannofossil assemblage of the matrix associated with CNHM 9350 contains rare and poorly preserved specimens. However, the presence, although very rare, of *Quadrum gartneri* and the absence of *Eiffellithus eximius* s.s., suggests the UC7 Zone (Burnett, 1999). The UC8 zone, however, cannot be ruled out because of the rare presence of *Lucianorhabdus*, which first appears just below the base of *E. eximius*. Hence, the data suggest a Turonian age for these specimens. This refutes the earlier hypothesis of a more recent age, i.e., Campanian (Murray et al., 2016) for CNHM 9350.

NHMUK PV P 9029a and NHMUK PV OR 39125 come from the Cenomanian lower Chalk (see Friedmann et al., 2016) of southern England (Woodward, 1889, 1893, 1912). NHMUK PV P 6524 and NHMUK PV P 51 come from the late Cenomanian *Holaster subglobosus* Zone (see Friedmann et al., 2016) of southern England (Woodward, 1889, 1893, 1912). Unfortunately, no information on the age of the other English specimens could be obtained. The isolated tooth MGL 6263 comes from the late Cenomanian *Actinocamax plenus* Zone (see Friedmann et al., 2016) of Upper France. See Table S1 in “Additional file 1” (supplementary material), for details of the specimens.

**Description of the new tooth plate.** Specimen CNHM 9350 (Figs. 4, 5) consists of a tooth set (75 teeth in total), which is still embedded in a matrix slab, and its counterpart (imprint; Fig. 4A, B). Among these teeth, 44 are still arranged with each other in their natural position, forming a dental plate (see Figs. 4, 5). In addition, 31 teeth together with several tooth fragments and dental imprints are scattered on the slab around the articulated dental plate (Figs. 4, 5). Most of the teeth display rectangular crowns crossed by 8–11 thin, occlusal ridges (e.g., Fig. 4C); the smallest teeth have a triangular (e.g., “3c” in Fig. 4D) or oval (e.g., “f1, g1” in Fig. 4E) shape and a lower number of ridges (four to six; see also Fig. 4D, E). The dental crown is bulgy in the teeth placed at the centre of the dental plate (Fig. 4F). Lateral teeth have a less high crown and those placed in the distal areas of the plate are completely flat (see Fig. 4F). Across the whole dental plate, the occlusal ridges on the teeth reach the lateral crown margins with their branching ends (Fig. 4C–E). Thin wrinkles and granules cover the anterior marginal area of the tooth crown. The central teeth are abraded on their occlusal surface especially at the anteriormost area of the plate (see Fig. 4A, C). The roots of the teeth are embedded in the matrix and, therefore, not observable.

**Redescription of the comparison material.** Specimens NHMUK PV OR 28348 (Fig. 6A, A^I^), NHMUK PV OR 28349 (Fig. 6B) and NHMUK PV OR 47904b (Fig. 6E) are isolated teeth with a rectangular, transversally elongated crown. Nine to eleven transversal ridges cross their occlusal crown surface. Their anterior margin shows no protuberance, while the posterior one has a shallow sulcus. Their occlusal surfaces are almost flat. The ridges branch at their ends, reaching the lateral margins of the crown. The ridges in NHMUK PV OR 28348 are damaged in the central area of the crown, while they exhibit a slight occlusal abrasion on the left side (see Fig. 6A^I^). The crown of NHMUK PV OR 47904b (Fig. 6E) is markedly abraded on the right side. The marginal area of NHMUK PV OR 28348 (Fig. 6A, A^I^), NHMUK PV OR 28349 (Fig. 6B) and NHMUK PV OR 47904b (Fig. 6A^I^) is covered by fine wrinkles on the anterior and posterior tooth margins. The tooth roots of NHMUK PV OR 28348 and NHMUK PV OR 28349 are not preserved. The two teeth associated in NHMUK PV OR 47904c (Fig. 6F) are almost identical to NHMUK PV OR 28348 (Fig. 6A^I^). Both teeth have eight occlusal ridges. The anteriormost ridges on one of the teeth in NHMUK PV OR 47904c (Fig. 6F) form a small loop on the right crown side. Specimens NHMUK PV OR 40541 (Fig. 6C) and NHMUK PV OR 47904a (Fig. 6D) are very similar to NHMUK PV OR 28348 (Fig. 6A^I^) and NHMUK PV OR 28349 (Fig. 6B), but their crown is squared and crossed by 13–15 ridges. The tooth roots of NHMUK PV OR 40541 and NHMUK PV OR 47904a–c still are embedded in matrix and therefore not accessible. Specimens NHMUK PV P 6524 (Fig. 6G–G^II^) and NHMUK PV P 51 (Fig. 6H–H^II^) are two small rectangular teeth with well-developed anterior protuberances and slightly raised occlusal surfaces. Specimen NHMUK PV P 6524 has a symmetrical outline (see Fig. 6G^I^). Most of the crown surface of NHMUK PV P 6524 and NHMUK PV P 51 is markedly abraded (see Fig. 6H^II^, G^II^). Fine ridges (?eight to nine) with branching ends are still recognisable on their occlusal surface. The marginal area is covered by fine wrinkles. Specimens NHMUK PV OR 39125a–d (Fig. 6I–L), NHMUK PV OR 28342 (Fig. 6N), NHMUK PV OR 49855 (Fig. 6Q), NHMUK PV P 1385 (Fig. 6S), NHMUK PV P 9029a (Fig. 6R) and NHMUK PV P 9718 (Fig. 6P) have squared crowns with rounded margins. The anterior protuberance is poorly developed and the posterior sulcus is shallow. Specimens NHMUK PV OR 39125a-d (Fig. 6I–L), NHMUK PV OR 28342 (Fig. 6N) and NHMUK PV P 9029a (Fig. 6R) display flat occlusal surfaces, while a bulgier crown characterizes NHMUK PV OR 49855 (Fig. 6Q), NHMUK PV P 1385 (Fig. 6S) and NHMUK PV P 9718 (Fig. 6P). Irregular, thin ridges (four to ten) with branching lateral ends cross the top tooth surface. The anteriormost ridges are quite transversal, while the rearmost ones are interrupted and branching on the posterior crown edge. The rearmost ridges of NHMUK PV OR 49855 (Fig. 6Q) form a sort of fold in the centre of the tooth crown. Thin wrinkles cover the marginal areas. Specimens NHMUK PV OR 39125e (Fig. 6M, M^I^) and NHMUK PV P 2688 (Fig. 6O–O^II^) have symmetrical and transversally elongated crowns similar to NHMUK PV P 6524 (Fig. 6G^I^) with seven to eight occlusal ridges. In lateral view (Fig. 6O^II^), the outline of the crown of NHMUK PV P 2688 is perpendicular to the tooth base, while it is tilted anteriorly. The ridges in NHMUK PV OR 39125e (Fig. 6M) and NHMUK PV P 2688 (Fig. 6O) branch posteriorly as in NHMUK PV OR 39125a–d (Fig. 6I–L). Specimens NHMUK PV OR 39125e and NHMUK PV P 2688 show a bulgy occlusal surface at the centre of their tooth crown (see Fig. 6M^I^, O^I^). Rare, coarse granules are randomly scattered between the central ridges and marginal wrinkles of NHMUK PV OR 39125a–e (Fig. 6I–M^I^) and NHMUK PV OR 28342 (Fig. 6N). In posterior view (Fig. 6J^I^), the tooth root of NHMUK PV OR 39125b is thick and bilobate with a shallow antero-posterior sulcus. The roots of NHMUK PV OR 39125a, NHMUK PV OR 39125c–e, NHMUK PV OR 28342, NHMUK PV OR 49855, NHMUK PV P 1385, NHMUK PV OR 5449, NHMUK PV P 9029a, NHMUK PV P 9718 and NHMUK PV P 2688 are not preserved or not accessible. Specimen MNHN.F.CTE221 (Fig. 7A–A^II^) is an isolated tooth with thirteen occlusal ridges very similar to those observed on NHMUK PV P 1385 (Fig. 6S), but its ornamentation lacks any posterior branching. In lateral view (Fig. 7A^II^), MNHN.F.CTE221 exhibits a bulgy dental crown tilted anteriorly; conversely, its posterior side is perpendicular to the tooth base. Specimen MGL 6263 (Fig. 7B–B^II^) has a rectangular and bulgy crown with seventeen occlusal ridges similar to those on the crown of NHMUK PV P 6524 (Fig. 6G^I^), NHMUK PV OR 39125e (Fig. 6M) and NHMUK PV P 2688 (Fig. 6O). None of the ridges of MGL 6263 branch posteriorly; the ridges are abraded at the centre of the occlusal surface (see Fig. 7B). Although the posterior corner of the right tooth edge in MGL 6263 is broken, its crown exhibits a quite regular and symmetric outline (see Fig. 7B). The tooth roots of MNHN.F.CTE221 and MGL 6263 are not preserved (see Fig. 7A^II^, B^II^).

#### Remarks.

All the specimens described here exhibit the typical ornamentation of *Ptychodus decurrens* Agassiz, 1838 with branching ridges on the edges of the tooth crown. However, their degree of branching can vary between teeth and / or between lateral margins of a single tooth. For examples, the branching at the end of the occlusal ridges is less pronounced on the left side in NHMUK PV OR 28348 (see Fig. 6A^I^) and on both sides in NHMUK PV OR 47904b–c (see Fig. 6F). The lower symphyseal teeth are well known to be the largest in any species of *Ptychodus* Agassiz, 1834. Conversely, small, narrow teeth characterise the upper dentition of both cuspidate and un-cuspidate species (see Amadori et al., 2020a, 2022a; Hamm, 2020; Shimada, 2012; Woodward, 1912). In particular, Woodward (1887) described an articulated dentition of *Ptychodus decurrens* Agassiz, 1838 preserving both upper and lower teeth still in natural position. The specimen clearly shows lower teeth characterised by bulgy crowns, while the upper ones have more flattened occlusal surfaces (see also Woodward, 1912: text fig. 76). This pattern of dignathic heterodonty was observed already in various other un-cuspidate species of *Ptychodus* (e.g., *P. latissimus*, *P. mediterraneus* and *P. polygyrus*; Amadori et al., 2020a, 2020b; Hamm, 2020; Woodward, 1912). The dental plate CNHM 9350 (Figs. 4, 5) clearly preserves several lower teeth of *Ptychodus decurrens* Agassiz, 1838 that are still arranged in natural position. This crushing plate was undoubtedly placed in the lower jaw due to the presence of the largest teeth placed across the symphyseal row (e.g., “m2–m6” in Fig. 4C). The first published drawings of isolated specimens of *P. decurrens* depict lower teeth probably belonging to the symphyseal (e.g., Fig. 1B, C) and lateral (e.g., Fig. 1A, D) rows. The type series of *P. decurrens* (Fig. 1E–L^I^) includes both lower (Fig. 1E, E^I^, J, J^I^, L, L^I^) and upper (Fig. 1K, K^I^) teeth. Among the isolated specimens redescribed here, the tooth NHMUK PV OR 5449 (Fig. 3) was originally placed within the symphyseal row of the lower dentition, based on its bulgy tooth crown with symmetrical outline (see also Woodward, 1889: p. 139). The bulgy, symmetrical teeth NHMUK PV P 6524 (Fig. 6G–G^II^), NHMUK PV OR 39125e (Fig. 6M, M^I^), NHMUK PV P 2688 (Fig. 6O–O^II^) and MGL 6263 (Fig. 7B–B^II^) belong to the same tooth row. The bulgy teeth NHMUK PV P 51 (Fig. 6H–H^II^), NHMUK PV P 9718 (Fig. 6P), NHMUK PV OR 49855 (Fig. 6Q), NHMUK PV OR 39125a–d (Fig. I–L), NHMUK PV OR 28342 (Fig. 6N), NHMUK PV P 1385 (Fig. 6S) and MNHN.F.CTE221 (Fig. 7A–A^II^) were originally arranged within lateral areas of the lower dental plate. Based on their asymmetrical crowns and flat occlusal surfaces, NHMUK PV OR 28348 (Fig. 6A–A^I^), NHMUK PV OR 28349 (Fig. 6B), NHMUK PV OR 40541 (Fig. 6C), NHMUK PV OR 47904a–c (Fig. 6D–F) and NHMUK PV P 9029a (Fig. 6R) are identified here as upper lateral teeth. In general, lateral teeth with a more transversally elongated crown (e.g., NHMUK PV OR 28348, NHMUK PV OR 28349, NHMUK PV OR 47904b-c) seem to be arranged close to the symphyseal row in un-cuspidate species (e.g., *P. decurrens* and *P. mediterraneus*; see Woodward, 1912: pl. 51, fig. 9; Amadori et al., 2020a: fig. 6).

Tooth abrasions observed on the lower crushing plate CNHM 9350 (see Fig. 5) indicate that the processing of prey items in *Ptychodus decurrens* Agassiz, 1838 occurred mostly at the centre of the dentition, as already hypothesised for other un-cuspidate species of *Ptychodus* (e.g., *P. mediterraneus*; see Amadori et al., 2020a). In particular, “functional teeth” (sensu Shimada, 2012) were limited to centre of the anterior portion of the crushing plates (see Amadori et al., 2020a). A similar tooth abrasion pattern also occurs in other articulated specimens of *P. decurrens* (e.g., Woodward, 1912: text fig. 76).

## Discussion

### Reconstruction of the complete lower tooth plate

The first reconstruction of a complete dentition of *Ptychodus decurrens* Agassiz, 1838 was attempted by Woodward (1904: text fig. on p. 134). Based on a small portion of an articulated tooth plate from the Upper Cretaceous of England, Woodward (1904) provided an interpretative sketch of the lower dentition of this un-cuspidate species (see also Woodward, 1912: text fig. 71). In Woodward’s reconstruction (Fig. 8A), roughly 115 teeth are arranged in one symphyseal row and five lateral rows for each hemiarch. Added to these are 22 “replacement teeth” (sensu Shimada, 2012) placed on the posterior margin of the dental plate and arranged mesiodistally in two files. The latter are folded and bend following the internal curvature of the oral cavity. The plate reconstructed by Woodward (1904) is elongated and narrow anteriorly (see Fig. 8A). As in most other species of *Ptychodus* (e.g., *P. maghrebianus*, *P. mediterraneus*, *P. mortoni* and *P. occidentalis*), the teeth decrease in size and bilateral symmetry mesiodistally across each hemiarch of the dentition (see also Amadori et al., 2020a, 2022a; Shimada, 2012; Shimada et al., 2009; Woodward, 1912). These general features are also confirmed by the reconstruction provided in the present study (Fig. 8B).

Differently from the previous reconstruction provided by Woodward (1904; see Fig. 8A), the lower dentition CNHM 9350 (Fig. 4A) shows eight lateral tooth rows (“l1–8” in Fig. 8B) and, consequently wider lateral hemiarches. The central antero-posterior rows (“m” and “l1–2” in Fig. 8B) are juxtaposed with the adjacent ones, while the more distal ones (“l3–8” in Fig. 8B) are slightly imbricated with each other. Teeth arranged in each single row do not decrease in size, which is in contrast to Woodward’s sketch, where anterior teeth were smaller than posterior teeth (see Fig. 8A). In the reconstruction presented here, the anterior shrinkage of the crushing plate is more gradual due to a higher number of lateral teeth (e.g., “l3–5” in Fig. 8B) reaching the anterior area of the dentition. The differences in Woodward’s reconstruction probably result from the poor preservation of the anterior part of the articulated specimen originally used (see Woodward, 1904: pl. 15, fig. 2). Conversely, the posterior part of the new dentition CNHM 9350 is poorly preserved (see Fig. 4A) and it is therefore impossible to identify replacement teeth. Based on the reconstruction of CNHM 9350 proposed here (Fig. 8B), the lower tooth plate has an estimate length of about 7 cm and a width of about 4.8 cm. The tooth plate comprises a total of 213 teeth excluding the replacement teeth. It has been estimated that replacement teeth account for around 12% of the entire lower dentition in various species of *Ptychodus* (see Shimada, 2012; Amadori et al., 2020a). If a similar proportion is applied to *P. decurrens*, the complete mandibular plate would have consisted of 242 teeth. According to this estimate, approximately 29 replacement teeth could be expected. Conversely, the reconstruction presented by Woodward (1904: text fig. on p. 134) shows two replacement teeth for each antero-posterior row. Following Woodward’s scheme, the replacement teeth in CNHM 9350 would amount to 34, giving a total of 247 teeth for the complete lower dentition.

Unfortunately, dental elements belonging to the upper dental plate are totally missing in CNHM 9350. Other known and new dentitions of *Ptychodus decurrens* (both lower and upper tooth plates) are currently under study and careful reassessment (Amadori et al., in prep.). Such a reappraisal will be important in order to further understand the morphology, heterodonty and function of the complete dentition (both upper and lower plates) of this durophagous predator.

### Comparison with similar taxa

*Ptychodus decurrens* Agassiz, 1838 is one of the best-known and most-recognisable species within the genus. The study of numerous sets of teeth (articulated and associated) allowed for a preliminary good characterisation of this taxon (e.g., Müller, 2008; Williamson, 1991; Woodward, 1887, 1904, 1912). In particular, the transversal ridges branching marginally, which are typical for *Ptychodus decurrens* Agassiz, 1838, are a rare ornamentation pattern among other species of *Ptychodus*. Nevertheless, the dentition of *P. decurrens* displays a marked intraspecific variability, as already observed in many other un-cuspidate taxa (e.g., *P. polygyrus*; Dibley, 1911; Woodward, 1912). Over the centuries, anomalous or somewhat ambiguous tooth morphologies observed in rare isolated teeth have led numerous authors to establish new, but dubious species. These are flanked by taxa that are actually similar to *P. decurrens* in tooth morphology, but certainly represent separate species.

***Ptychodus decurrens *Agassiz,** 1838**vs** ***Paraptychodus washitaensis*** **Hamm,** 2015**.** Hamm (2015) describe a new genus (*Paraptychodus*) of ptychodontid sharks based on isolated teeth from the Albian of Texas (USA). The type species, *P. washitaensis*, exhibits rectangular to triangular tooth crowns crossed by transversal ridges; the ridges bifurcate and branch out reaching the lateral margins of the occlusal surface (Hamm, 2015: figs. 2, 3). Although the general shape and ornamentation of the tooth crown are reminiscent of those described for *Ptychodus decurrens*, the morphology of the tooth root of *Paraptychodus washitaensis* is markedly different. In addition to the rectangular shape and the two lobes typical for tooth roots of all species within the genus *Ptychodus* (e.g., Amadori et al., 2019a, 2020a, 2022a; Hamm, 2020; Shimada et al., 2009; Woodward, 1912), the roots in *Paraptychodus washitaensis* shows a protuberance on the posterior face never seen in any other ptychodontid shark (see Hamm, 2015: figs. 2, 3). In addition, distal (“posterior” sensu Hamm, 2015) teeth of *P. washitaensis* have an almost pointed occlusal surface (see Hamm, 2015: fig. 4), whereas the latter is flat in distal teeth of *Ptychodus decurrens*. Therefore, *Ptychodus* and *Paraptychodus* have to be considered separate taxa.

***Ptychodus decurrens *Agassiz,** 1838**vs** ***P. depressus*** **Dixon,** 1850**.** Based on an isolated tooth (NHMUK PV OR 28348; see Fig. 6A, A^I^) with flat occlusal surface, Dixon (1850: p. 365, pl. 31, fig. 9) described a new species *Ptychodus depressus*. However, the occlusal ornamentations of this specimen is identical to those of the upper teeth arranged within the articulated dentition of *P. decurrens* documented by Woodward (1887: pl. 10, figs. 4–10). Moreover, Dixon (1850: p. 365) himself noticed the morphological similarities between the large, flat teeth of *P. depressus* and *P. polygyrus*. Although *P. polygyrus* has a very different ornamentation, the crown in its upper teeth is quite flattened. Dignathic heterodonty with bulgy lower teeth and almost flat upper teeth indeed was documented for various un-cuspidate species of *Ptychodus* (e.g., *P. decurrens*, *P. mediterraneus* and *P. polygyrus*; Amadori et al., 2019a, 2020a; Woodward, 1887, 1912). The morphological differences between the teeth assigned to *P. depressus* and *P. decurrens* are here attributed to the marked dignathic heterodonty commonly observed in *Ptychodus* taxa. For all the aforementioned reasons, the species *P. depressus* has to be considered as junior synonym of *P. decurrens* (see also Woodward, 1912: p. 239). Conversely, the occlusal ornamentation patterns of *P. depressus* (= *P. decurrens*) and *P. polygyrus* clearly indicate that they belong to different species.

***Ptychodus decurrens *Agassiz,** 1838** vs** ***P. levis*** **Woodward,** 1893. Woodward (1893: p. 192, pl. 5, fig. 5, 6) described two small, isolated teeth (NHMUK PV P 51 and NHMUK PV P 6524; Fig. 6G–H^II^) from southern England assigning them to a new species named *Ptychodus levis*. The teeth of *P. levis* and *P. decurrens* are very similar to each other in both general shape and ornamentation, as already was noted by Woodward (1893). The lack of occlusal abrasions mentioned by Woodward (1893: p. 192) in the original description does not match with what was observed in the specimens of the present study. Clearly, the crown of both teeth is markedly worn (see Fig. 6G^II^, H^II^). Similar abrasions were described for various species of *Ptychodus* and related to prey processing (e.g., Amadori et al., 2019a, 2019b, 2020a, b; Shimada, 2012). The marked occlusal tooth wear of NHMUK PV P 51 and NHMUK PV P 6524 masked the typical pattern of *P. decurrens* and turned it in “very feeble transverse ridges and furrows, passing gradually at the extremities into the still more delicate striations of the marginal area” used by Woodward (1893: p. 192) as diagnostic characters for his new species, *P. levis*. Later, Woodward (1912: p. 239) revised his position and synonymised *P. levis* and *P. decurrens*. Furthermore, small teeth (e.g., NHMUK PV P 51 and NHMUK PV P 6524) may have less marked ornamentations than larger, more massive ones. For the above reasons, the taxon *P. levis* has to be regarded as junior synonym of *P. decurrens*.

***Ptychodus decurrens *Agassiz,** 1838** vs** ***P. multistriatus*** **Woodward,** 1889**.** Based on an associated tooth set (NHMUK PV P 2681) composed of seven teeth, Woodward (1889: p. 146, pl. 5, fig. 4–6) established the new species *Ptychodus multistriatus*. Woodward (1889: p. 146) described the teeth of *P. multistriatus* as “very similar to those of *P. polygyrus* but having the transverse ridges upon the crown relatively much more delicate and numerous”. Specimen NHMUK PV P 2681 shows irregular ridges, partial loops and no branching at their lateral ends (see Woodward, 1889: pl. 5, figs. 4–6). Later, Woodward (1912: p. 239) changed his mind and synonymised *P. multistriatus* with *P. decurrens* due to the presence of partial loops that can occur in the occlusal ornamentation of both species (e.g., Amadori et al., 2023; Woodward, 1912). Despite this, the lack of ridges with branching ends in *P. multistriatus* is here considered sufficient and crucial in order to keep this taxon separate from *P. decurrens*. A possible, and even probable, synonymy between *P. multistriatus* and *P. polygyrus* remains pending until further and more detailed comparisons between the type specimens of the two taxa have been conducted.

***Ptychodus decurrens *Agassiz,** 1838** vs *P. oweni*** **Dixon,** 1850**.** Dixon (1850: p. 365) established the species *P. oweni* based on a set originally including eight to ten associated teeth. Only one of them (see Fig. 6I, I^I^) was already described and figured by Dixon (1850: pl. 31, fig. 2). Unfortunately, only four teeth from the original set (NHMUK PV OR 39125) were identified (see Fig. 6I–M^I^), while the others are probably lost. The occlusal ornamentation pattern of the tooth originally figured by Dixon (1850) with irregular and almost radial branching ridges is quite different from the typical transversal arrangement of the ridges in *P. decurrens*. However, a transition of the ridge pattern from “near-radial” (e.g., Fig. 6I, I^I^) to “transversal” (e.g., Fig. 6M, M^I^) can be observed among the teeth belonging to the tooth set NHMUK PV OR 39125 (see also Woodward, 1912: p. 243). These anomalous ornamentations are thus considered here to be part of the intraspecific variability of *P. decurrens*. The reasons for these radial and irregular occlusal patterns in *P. decurrens* are still unknown. Due to the transitional morphologies present in the type series of *P. oweni* and the lack of other occurrences with such anomalous ornamentations worldwide, the species *P. oweni* should be considered a junior synonym of *P. decurrens* (see also Woodward, 1912: p. 239).

***Ptychodus decurrens *Agassiz,** 1838** vs *P. occidentalis *Leidy,** 1868**.** *Ptychodus occidentalis* has been established based on seven isolated teeth (ANSP 1460–1466; see Leidy, 1868: p. 207, pl.17, fig. 7, 8, pl. 18, fig. 15–18). All teeth of this type series exhibit occlusal ornamentations similar to those of *P. decurrens*. Among them, there are also small specimens possibly belonging to other species (e.g., *P. decurrens*; see Leidy, 1868: p. 207). However, a blunt cone with a “knob-like” apex covers most of the occlusal surface of the largest tooth of *P. occidentalis* (ANSP 1465; see Hamm, 2020: fig. 28A1–A3). This protuberance is narrower and higher than the raised and massive crown of the lower teeth of *P. decurrens*. The tooth cusp in *P. occidentalis* protrudes forward in most teeth, while the tooth crown in *P. decurrens* is typically raised at the rear with a lateral outline tilted anteriorly. Furthermore, the articulated specimen of *P. occidentalis* described by Shimada et al. (2009) clearly shows that conical cusps characterise all teeth within both its upper and lower dental plates. Differently, almost flat teeth compose the upper plate and the more distal areas of the lower plate in *P. decurrens* (see also Woodward, 1887, 1912). Despite the undeniable similarities between the dentitions of *P. occidentalis* and *P. decurrens*, these have to be considered as separate species.

***Ptychodus decurrens *Agassiz,** 1838** vs *P. rhombodus*** **Underwood & Cumbaa,** 2010**.** Based on an isolated tooth (RSM P 2989.51), Underwood and Cumbaa (2010) established the new species *Ptychodus rhombodus*. The holotype has an asymmetrical shape and a raised occlusal surface with ridges branching at their lateral ends (see Underwood & Cumbaa, 2010: pl. 2, figs. 1, 2). The general morphology and occlusal ornamentations of the teeth assigned to *P. rhombodus* are very reminiscent of those observed in *P. decurrens*. However, the holotype of *P. rhombodus* has a raised crown protruding anteriorly (see Underwood & Cumbaa, 2010: pl. 2, figs. 2), which makes it more similar to the asymmetrical, lateral teeth of *P. occidentalis* (see Shimada et al., 2009: fig. 5B, C, F). The occlusal cusp also is characteristic of *P. anonymus* (see Hamm, 2020), but *P. rhombodus* differs from the latter in having branching ridges (see Underwood & Cumbaa, 2010). Indeed, the occlusal ridges of *P. anonymus* form lateral loops with concentrical arrangement (see Hamm, 2020: figs. 21–24). Together with the holotype, more than 150 other isolated teeth were assigned to *P. rhombodus* (see Underwood & Cumbaa, 2010). Among these additional specimens, some teeth have a dome-shaped occlusal surfaces, which are more raised posteriorly (see Underwood & Cumbaa, 2010: pl. 2, figs. 5, 6). These actually might belong to *P. decurrens*. Due to the presence of a central, forward protruding “cusp” in the holotype of *P. rhombodus* (Underwood & Cumbaa, 2010: p. 910), this species is considered here to be separate from *P. decurrens*. The possible synonymy between *P. rhombodus* and *P. occidentalis* remains pending until more detailed studies and comparisons of the type material of these two species have been conducted.

**Varieties and subspecific ranks.** The term “variety” is currently used below the species level in animal and plant classification to identify a group of individuals that differ distinctly from each other, but still can interbreed with other varieties of the same species (see Martin & Hine, 2015). Applying such a definition is obviously impossible in a palaeontological context, where taxa (e.g., morphospecies) are commonly identified on a morphological basis only (e.g., Cappetta, 2012). According to the ICZN (1999: art. 10.2 and art. 45.6.3 and “Glossary” section), terms such as “varieties” or “forms” denote infrasubspecific ranks, and therefore are not available, if published after 1960. Conversely, any scientific name published before 1961 for which its author expressly used the terms “variety” or “form” must be regarded as subspecific rank (available; see ICZN, 1999: art. 45.6.4).

In addition to various cuspidate and un-cuspidate species, the marked morphological variability characterizing the fossil dentitions of *Ptychodus* spp. also has prompted several authors to establish dubious “varieties” within species based on isolated teeth. Agassiz (1839) described several isolated teeth as belonging to *Ptychodus polygyrus* Agassiz, 1835, but subdivided them into four “variétés” (varieties; see Amadori et al., 2020a; Brignon, 2019). Two teeth figured by Agassiz (1838: pl. 25b, fig. 21; 1839: pl.25, fig. 9) were assigned to “*P. polygyrus* var. *sulcatus*” (Agassiz, 1839: p. 156). In particular, one of these two isolated teeth (MNHN.F.CTE221; see Fig. 7A–A^II^) exhibits the species-specific morphology of *P. decurrens* Agassiz, 1838. The other one shows features similar to the typical teeth of *P. polygyrus* Agassiz, 1835 (see also Amadori et al., 2020a). Leriche (1902: p. 96–97, t. 2, fig. 20) also established *P. decurrens* var. *multiplicatus* as a new variety within the species *P. decurrens* based on a large tooth (see Fig. 7B–B^II^) with fine ornamentation from the Cenomanian of Autreppes (Upper France). Again, there are no significant differences between the holotype of this second variety and the typical morphology of *P. decurrens*. Consequently, the tooth MNHN.F.CTE221 previously identified as *P. polygyrus* var. *sulcatus* Agassiz, 1839 and the tooth MGL 6263 originally described as *P. decurrens* var. *multiplicatus* Leriche, 1902 are reassigned here to the species *P. decurrens*. Conversely, the other tooth assigned to *P. polygyrus* var. *sulcatus* by Agassiz (1839) probably belong to *P. polygyrus* (see also Amadori et al., 2020a).

## Conclusions

During the Turonian species diversity peak of *Ptychodus*, the spatial range of the un-cuspidate *Ptychodus decurrens* within the peri-Tethyan seas reached the southern area of the Adriatic carbonate platform. The Turonian age established here for the newly described lower dentition of *P. decurrens* backdates the Upper Cretaceous outcrops of the Prapatnica area (Dalmatia, southern Croatia), previously assumed to be of Campanian age. Although *P. decurrens* is considered a low-crowned species, its lower dentition has bulgy (“high-crowned”) teeth limited to the centre of the crushing plate. This is a common feature in un-cuspidate species of *Ptychodus*. Therefore, not all teeth belonging to un-cuspidate taxa show a “low-crowned” morphology. However, in all dentitions of un-cuspidate *Ptychodus*, tooth cusps are absent. The terms high- and low-crowned are thus not equivalent to cuspidate and un-cuspidate, respectively. In order to prevent confusion, we propose here to limit the use of high- and low-crowned only to describe tooth morphologies. Conversely, the terms cuspidate and un-cuspidate can also be applied to identify dental remains of *Ptychodus* at species level.

The morphology of the tooth root is useful for distinguishing fossil shark genera (e.g., *Paraptychodus* and *Ptychodus*) within the family Ptychodontidae. *Ptychodus decurrens* exhibits very clear species-specific characters consisting of branching ridges covering most of the occlusal surface. These characters are well identifiable in the symphyseal tooth belonging to the original type series that was selected here as lectotype. Nevertheless, the direction of the aforementioned occlusal ramifications may vary involving even the posterior part of the dental crown. In view of an increasingly clear and stable taxonomy and systematics of fossil elasmobranchs, the identification of unambiguous characters indicative for taxonomic purposes is essential. Morphological features observed in isolated, disarticulated specimens alone can lead to misinterpretation and confusion, artificially increasing the number of species. When possible, a more rigorous and modern approach based on detailed comparisons of articulated dentitions is desirable to ensure solid and successful progress in the study of species of *Ptychodus*, as well as for fossil elasmobranchs in general. The present reassessment based on both type material and an exceptionally well-preserved dentition of *P. decurrens* clarifies the taxonomic status of one of the most iconic and geographically widespread species of Late Cretaceous ptychodontid sharks.

## Competing interests

The authors declare no competing interests.

## Supplementary Information

**Additional file 1:** Table S1. Summary of the (Is, isolated; As, associated; Ar, articulated) material from the Upper Cretaceous (Cen, Cenomanian; Tur, Turonian) examined and assigned here to *Ptychodus decurrens* Agassiz, 1838. The asterisk indicates the new dentition from Dalmatia (Croatia). L, selected lectotype; TM, specimens originally described as type material.

**Additional file 2:** Extended synonyms list for the species *Ptychodus decurrens* Agassiz, 1838 and additional list of references.

## Data Availability

All data generated or analysed during this study are included in this published article (and its supplementary information files).
