# Supplementary material for: New insights on the shell-crusher shark Ptychodus decurrens Agassiz, 1838 (Elasmobranchii, Ptychodontidae) based on the first known articulated dentition from the Upper Cretaceous of Croatia
Source: Swiss J Palaeontol. 2025 Jan 8;144(1):2. doi: 10.1186/s13358-024-00340-7 (PMC11711565; doi:10.1186/s13358-024-00340-7)
Supplement: Supplementary file 1 — Additional file 1: Table S1. Summary of the (Is, isolated; As, associated; Ar, articulated) material from the Upper Cretaceous (Cen, Cenomanian; Tur, Turonian) examined and assigned here to Ptychodus decurrens Agassiz, 1838. The asterisk indicates the new dentition from Dalmatia (Croatia). L, selected lectotype; TM, specimens originally described as type material. [file 13358_2024_340_MOESM1_ESM.docx]

Swiss Journal of Palaeontology

**SUPPLEMENTARY MATERIAL**

**New insights on the shell-crusher shark *Ptychodus decurrens* Agassiz, 1838 (Elasmobranchii, Ptychodontidae) based on the first known articulated dentition from the Upper Cretaceous of Croatia**

Manuel Amadori^1*^, Sanja Japundžić^2^, Jacopo Amalfitano^3^, Luca Giusberti^3^, Eliana Fornaciari^3^, Patrick L. Jambura^1,4^ and Jürgen Kriwet^1,4^

**Additional file 2:**

**Table S1.** Summary of the (Is, isolated; As, associated; Ar, articulated) material from the Upper Cretaceous (Cen, Cenomanian; Tur, Turonian; ?, unknown) examined and reassigned here to *Ptychodus* *decurrens* Agassiz, 1838. The asterisk indicates the new dentition from Dalmatia (Croatia). L, selected lectotype; TM, specimens originally described as type material.

| **Specimen** | **Original identification** | **Description** | **Locality of provenance** | **Age** |
| --- | --- | --- | --- | --- |
| CNHM 9350* | *P. decurrens* | Lower teeth (Ar) | Prapatnica, Dalmatia, Croatia | Tur |
| MGL 6263 (TM) | *P. decurrens* var. *multiplicatus* | Symphyseal tooth (Is) | Autreppe, Upper France | Cen |
| MHNN FOS. 474 (TM) | *P. decurrens* | Lower tooth (Is) | England | ? |
| MNHN.F.CTE221 | *P. polygyrus* var. *sulcatus* | Lateral tooth (Is) | England | ? |
| NHMUK PV OR 28342 | *P. oweni* | Lateral tooth (Is) | Lewes, East Sussex, England | ? |
| NHMUK PV OR 28348 (TM) | *P. depressus* | Upper tooth (Is) | Sussex, UK | ? |
| NHMUK PV OR 28349 | *P. decurrens* var. *depressus* | Upper tooth (Is) | Lewes, East Sussex, England | ? |
| NHMUK PV OR 40541 | *P. depressus* | Upper tooth (Is) | Maidstone, Kent, England | ? |
| NHMUK PV OR 47904 | *P. depressus* | Four lateral teeth (As) | Maidstone, Kent, England | ? |
| NHMUK PV OR 49855 | *P. decurrens* var. *oweni* | Lateral tooth (Is) | Glynde, East Sussex, England | ? |
| NHMUK PV OR 39125 (TM) | *P. oweni* | Five teeth (As) | Halling, Kent, England | Cen |
| NHMUK PV P 51 (TM) | *P. levis* | Lateral tooth (Is) | Dover, Kent, England | Cen |
| NHMUK PV P 1385 | *P. decurrens* var. *oweni* | Lateral tooth (Is) | Kent, England | ? |
| NHMUK PV P 2688 | *P. oweni* | Upper tooth (Is) | Sussex, England | ? |
| NHMUK PV OR 5449 (TM, L) | *P. decurrens* | Lower lateral tooth (Is) | Lewes, East Sussex, England | ? |
| NHMUK PV P 6524 (TM) | *P. levis* | Lateral tooth (Is) | Burham, Kent, England | Cen |
| NHMUK PV P 9029a | *P. decurrens* var. *oweni* | Lateral tooth (Is) | Burham, Kent, England | Cen |
| NHMUK PV P 9718 | *P. decurrens* var. *oweni* | Lateral tooth (Is) | England | ? |
