# Supplementary material for: New insights on the shell-crusher shark Ptychodus decurrens Agassiz, 1838 (Elasmobranchii, Ptychodontidae) based on the first known articulated dentition from the Upper Cretaceous of Croatia
Source: Swiss J Palaeontol. 2025 Jan 8;144(1):2. doi: 10.1186/s13358-024-00340-7 (PMC11711565; doi:10.1186/s13358-024-00340-7)
Supplement: Supplementary file 2 — Additional file 2: Extended synonyms list for the species Ptychodus decurrens Agassiz, 1838 and additional list of references. [file 13358_2024_340_MOESM2_ESM.docx]

Swiss Journal of Palaeontology

**SUPPLEMENTARY MATERIAL**

**New insights on the shell-crusher shark *Ptychodus decurrens* Agassiz, 1838 (Elasmobranchii, Ptychodontidae) based on the first known articulated dentition from the Upper Cretaceous of Croatia**

Manuel Amadori^1*^, Sanja Japundžić^2^, Jacopo Amalfitano^3^, Luca Giusberti^3^, Eliana Fornaciari^3^, Patrick L. Jambura^1,4^ and Jürgen Kriwet^1,4^

**Additional file 2:**

Extended synonyms list for the species *Ptychodus decurrens* Agassiz, 1838 and additional list of references.

Systematic Palaeontology

Class **Chondrichthyes** Huxley, 1880

Subclass **Elasmobranchii** Bonaparte, 1838

Order **Lamniformes** Berg 1937

Family **Ptychodontidae** Jaekel, 1898

Genus ***Ptychodus*** Agassiz, 1834

***Ptychodus decurrens*** Agassiz, 1838

Complete synonymic list:

p.1752 *Dentem seu palatum piscis Ostracionis*; Bruckmann: p. 116; pl. 5, fig. 4 (non fig. 3).

p.1752 “ipsum dentem exemtum petrefactum”; Bruckmann: p. 120; pl. 6, fig. 4.

1811 “unknown fish”; Parkinson, p. 262; pl.18, fig. 12.

1832 “Appareil dentaire d’un poisson”; Passy, pl. 15, fig. 4.

*1835* *Ptychodus decurrens* Ag.; Agassiz: p. 54 (*nomen nudum*).

*vp.1838 *Ptychodus decurrens* Agass.; Agassiz: atlas vol. 3, pl. 25b, figs. 1, 6-8 (non figs. 3-5).

vp.1838 *Ptychodus polygyrus* Agass.; Agassiz: atlas vol. 3, pl. 25b, fig. 21 (non figs. 4-8, 10, 11).

1839 *Ptychodus decurrens* Agass.; Agassiz: vol. 3; p. 154.

vp.1839 *Ptychodus polygyrus* Agass.; Agassiz: atlas vol. 3, pl. 25, fig. 9 (non figs. 4-8, 10, 11).

?1845 *Pt. decurrens* Ag.; Reuss; p. 1, pl.2, figs. 9, 10.

(1845) *Ptychodus decurrens*; Owen: atlas vol. 1, pl. 18, pl. 19, figs. 1, 2.

v1850 *Ptychodus decurrens*; Dixon: p. 362; pl. 30, fig. 7, 8; pl. 31, fig. 1; pl. 32, fig. 5.

v1850 *Ptychodus depressus* new; Dixon: p. 363; pl. 31, fig. 9.

v1850 *Ptychodus* *Oweni* new; Dixon: p. 364; pl. 31, fig. 2.

1852 *Ptychodus decurrens*; Gervais: atlas, pl. 78, fig. 5,5a.

1852 *Ptychodus decurrens* Ag.; Kiprijanoff: p. 487; pl. 13, figs. 4A-C, 5A-D.

1861 *P. decurrens* Ag.; Rogovich: p. 14; pl. 2, figs. 1,1a, 2.

(1875a) *Pt. decurrens* Ag.; Geinitz: p. 296; pl. 64, figs. 24, 25.

(?)1878 *Ptychodus latissimus* Agassiz; Zareczny: p. 200, pl. 8, fig. 8.

1878 *Ptychodus decurrens* Ag.; Fritsch: p. 14; fig. 34.

p.1885 *Pt. decurrens*; Quenstedt: p. 282; pl. 21, figs. 63, 64 (non figs. 61, 62)

1887 *Ptychodus* sp.; Woodward: pl. 10, fig. 1.

1887 *P. decurrens*; Woodward: p. 123; pl. 10, figs. 2-10, 13.

1889 *Ptychodus oweni* Dixon; Woodward: p. 138; pl. 5, fig. 8.

1890 *Ptychodus decurrens* Ag.; Zittel: p. 26, text fig. 24, p. 79, text fig. 78.

(?)1890 *Ptychodus polygyrus* Ag.; Zittel: p. 78, text fig. 77.

1890 *Ptychodus decurrens* Agass.; Davis: p. 376; pl. 38, figs. 1, 2.

1893 *Ptychodus levis,* sp. nov.; Woodward: p. 192; pl. 5, figs. 5, 6.

1900 *Ptychodus decurrens* Agassiz; Seguenza: p. 476; pl. 5, fig. 20.

1902 *Ptychodus decurrens* Agassiz; Leriche: p. 95; pl. 2, fig. 19.

1902 *Ptychodus decurrens* var. *multiplicatus* nov. var.; Leriche: p. 96; pl. 2, fig. 20.

1902 *Ptychodus polygyrus* Ag.; Zittel: p. 43, text fig. 85.

1902 *Ptychodus decurrens* Ag.; Zittel: p. 43, text fig. 86.

1904 *Ptychodus decurrens*; Woodward: p. 133; text fig., p. 134; pl. 15, figs. 1-5.

1904 *Ptychodus latissimus* Ag.; Squinable; p. 8; pl. 1, fig. 1.

*1905* *P. decurrens* Ag.; Pantanelli: p. 37.

1905 *Pt. decurrens*; Sacco: p. 254; pl. 8, fig. 14.

*1906* *Ptychodus decurrens* L. Agassiz; Leriche: p. 55.

*1907* *Ptychodus decurrens* Ag.; Jacob: p. 307.

*1909* *Pt. decurrens*, Ag.; Limanowski: p. 18.

1910 *Ptychodus decurrens* Agas.; Canestrelli: p. 117; pl. 1, figs. 1, 1a, 6-10.

p.1911 *Ptychodus decurrens* Agassiz.; Dibley: p. 264; pl. 17, fig. 2; pl. 19, figs. 20-24 (non figs. 9, 11, 13, 19).

1911 *Ptychodus decurrens* var. *depressus* (Dixon); Dibley: p. 266; pl. 17, figs. 1a, b.

1911 *Ptychodus decurrens* var. *levis* (A. S. Woodward); Dibley: p. 267.

1911 *Ptychodus decurrens* var. *oweni* (Dixon); Dibley: p. 266; pl. 18, figs. 1-11; pl. 19, figs. 1-5, 8, 15.

1911 *Ptychodus polygyrus* var. *sulcatus* (Agassiz); Dibley: p. 270.

*1912* *Ptychodus decurrens* Ag.; Priem: p. 264.

1912 *Ptychodus decurrens* Agassiz; Woodward: p. 239; text fig. 70; text fig. 71; text fig. 76, text fig. 77; pl. 51, pl. 52 (non syn.).

v1922 *Ptychodus decurrens* Ag.; D’Erasmo: p. 17; pl. 2, fig. 7, 8.

1951 *Ptychodus* cf. *martini*; Reinhart: p. 196; pl. 6, fig. 1.

1953 *Ptychodus decurrens* L. Agassiz; Casier: p. 6; text fig. 2B.

1958 *Ptychodus decurrens* Agass.; Berg: p. 73, text fig. 50.

1966 *Ptychodus decurrens*; Patterson: p. 340, text fig. 30A.

1966 *Ptychodus* *decurrens* var. *oweni* Dixon; Patterson: p. 344, text fig. 30B.

*1972 P. decurrens* Agassiz; Kauffman: p. 440.

v1976 *Ptychodus decurrens* Agassiz 1839; Sorbini: p. 482; pl. 5, figs. IG 37471-IG 37482.

v1976 *Ptychodus polygyrus* Agassiz 1839; Sorbini: p. 483; pl. 4, fig. IG 37483.

1977 *Ptychodus decurrens* Agassiz L. 1835; Herman: p. 49; text fig. on p. 52, pl. 2, fig. 1 (non syn.).

1977 *Ptychodus oweni* Dixon F. 1850; Herman: p. 53; text fig., p. 53.

1983 *Ptychodus decurrens* Agassiz; Marcinowski & Radwanski: pl. 8, figs. 8-12.

1987 *Ptychodus decurrens* Agassiz; Longbottom & Patterson: p. 255; text-fig. 11.3; pl. 55, fig. 1.

1987 *Ptychodus oweni* Dixon; Longbottom & Patterson: p. 255; pl. 55, fig. 2.

1991 *Ptychodus decurrens*; Williamson et al.: p. 595; text fig. 2, pl.1, fig. 1-14.

1992 *Ptychodus decurrens*; Hoch: p. 278; fig. 2.

1993 *Ptychodus decurrens* Agassiz 1835; Williamson et al.: p. 450, fig. 3.5-7.

1993 *Ptychodus decurrens* Agassiz 1843; Welton & Farish: p. 59; text fig. 1-7, p. 59.

1994 *Ptychodus decurrens* Agassiz; Astolfi & Colombara: p. 34; text figs. on p. 34, 35.

p.1996 *Ptychodus decurrens* Agassiz, 1839; Radwńanski & Marcinowski: pl. 2, fig. 1 (non fig. 3).

1999 *Ptychodus decurrens* Agassiz, 1843; Cappetta & Case; p. 11; pl. 2, figs. 7-9.

1999 *Ptychodus decurrens* Agassiz 1839; Siverson: p. 50; fig. 6.2.

2001 *Ptychodus decurrens* Agassiz, 1843; Cicimurri: p. 31; fig. 6C, D.

2002 *Ptychodus decurrens* Agassiz, 1838; Antunes & Cappetta: p. 103; pl.1, fig. 1-2.

2003 *Ptychodus decurrens* Agassiz; Astolfi & Colombara: p. 145; text fig. 206.

*2004 P. decurrens*; Dutheil & Ackermann: p. 1.

2004 *Ptychodus decurrens* Agassiz 1839; Mendiola: p. 3; fig. 2.

p.2004 *Ptychodus* aff. *mammillaris* Agassiz 1839; Mendiola: p. 3; pl. 1, figs. 2-4 (non fig. 1) (non syn.).

2005 *Ptychodus*; Dalla Vecchia et al.: p. 106; text fig. 79.

2006 *Ptychodus decurrens* Agassiz, 1839; Alvarado-Ortega et al.: p. 264; fig. 3B.

2006 *Ptychodus decurrens* Agassiz, 1835-39; Cumbaa et al.: p. 142; figs. 4.5, 4.6, 10.

2006 *Ptychodus decurrens* Agassiz, 1843; Shimada et al.: p. 142; figs. 6.3, 6.4.

2008 *Ptychodus decurrens*; Cuny: p. 112; fig. 1D.

2008 *Ptychodus decurrens* Agassiz; Shimada & Martin: p. 91; figs. 4B.

2008 *Ptychodus decurrens* Agassiz, 1835; Müller: p.62; pl. 1, 2.

2008 *Ptychodus* sp.; Rindone: p. 60; fig. 5b, c.

2009 *P. decurrens* Agassiz, 1843; Shimada et al.: p. 340; fig. 7A.

2009 *Ptychodus decurrens* (Agassiz, 1835); Carrillo-Briceño: p. 208; fig. 2.

2009 *Ptychodus occidentalis*; Lucas & Spielmann: p. 312; fig. 2E-I.

2009 *Ptychodus* sp.; Bravo-Cuevas et al.: p. 158; fig. 5F.

2009 *Ptychodus* sp.; González-Rodríguez & Fielitz: p. 69; text fig. 3B.

p. 2009 *Ptychodus latissimus* Agassiz 1843; Hamm: p. 4; fig. 2A, C, D (non fig. 2B, E-R).

2010 *Ptychodus decurrens*; Cumbaa et al.: p. 205; fig. 3G.

2010 *Ptychodus* ex. gr. *decurrens* Agassiz, 1838; Underwood & Cumbaa: p. 911; pl. 3, figs. 1-6.

p. 2010 *Ptychodus rhombodus* sp. nov.; Underwood & Cumbaa: p. 910; pl. 2, figs. 5, 6 (no pl. 2, figs. 1-4, 7-17; pl. 8, fig. 1).

*2012* *P. oweni* Dixon 1850A; Cappetta: p. 81.

2012 *Ptychodus decurrens*; Diedrich: p. 253; fig. 8C.

2012 *Ptychodus decurrens* Agassiz 1838 (1843B); Cappetta: p. 81; fig. 66A-E.

2012 *Ptychodus decurrens* Agassiz 1843; Verma: p. 184; fig. 2, 3.

p.2013 Ptychodus decurrens Agassiz 1835; Diedrich: p. 23; figs. 5A, 5B, 5D, 6.3, 6.5, 6.8-6.10, 9.2 (non fig. 6.1, 6.2, 6.4, 6.6, 6.7).

2013 *P. oweni*; Diedrich: p. 23; fig. 9.1.

2013 *Ptychodus mammilaris* (sic) Agassiz, 1838; Guinot et al.: p. 593; fig. 3E, F.

2013 *Ptychodus rhombodus*; Gallardo et al.: p. 113; fig. 3B.

2014 *Ptychodus decurrens* Agassiz, 1838; Vullo & Courville: p. 195; fig. 2A.

*2015 Pt. decurrens*; Brignon: p. 6.

2015 *P. oweni*; Jagt-Yazykova & Jagt: p. 33; fig. 4.2.

2015 *Ptychodus decurrens*; Jagt-Yazykova & Jagt: p. 33; fig. 4.1.

2015 *Ptychodus decurrens* Agassiz, 1843; Verma: p. 57; fig. 3m, n.

p.2016 *Ptychodus* sp.; Hoffman: p. 743; figs. 3.7-9, 14 (non figs. 3.1-6, 7-13)

2017 *Ptychodus decurrens*; Fischer et al.: p. 14; fig. 2a.

2018 *Ptychodus decurrens* Agassiz, 1839; Biryukov: p. 30; pl. 2, fig. 2.

2018 *Ptychodus decurrens* Agassiz 1839; Capasso: p. 42; fig. 1.

2018 *Ptychodus* sp.; López-horgue et al.: p. 67; fig. 2G.

2019 *Ptychodus decurrens* Agassiz, 1838; Amadori et al.: p. 230; text figs. 1D, 6, pl. 2.

2019 *Ptychodus decurrens* Agassiz, 1838; Brignon: p. 6; figs. 1, 5C.4,D.4, 8B.5, 8D, 18C, 44, 51C (middle fig.), 52B-E, 54 (bottom fig., p. 70), 57 (three middle figs.), 58A,B.

2020 *P. levis* Agassiz 1835; Hamm: p. 28; fig. 49.

2020 *Pt. decurrens* Agassiz, 1838; Brignon: p. 453; fig. 26A, B, D.

2020 *Ptychodus decurrens*; Hoyez et al.: p. 80; pl. 38, figs. 14, 19.

2020 *Ptychodus decurrens* Agassiz 1835; Hamm: p. 26; figs. 37-48.

2020 *Ptychodus decurrens* Agassiz, 1838; Amalfitano et al.: p. 267; fig. 6.

2020 *Ptychodus oweni* Dixon 1850; Hamm: p. 29; fig. 50.

2020 *Ptychodus sp.*; Hoyez et al.: p. 212; pl. 38, figs. 15-18, 20.

v2022 *Ptychodus decurrens* Agassiz, 1838; Amadori et al.: p. 9, fig. 5.

v2023 *Ptychodus decurrens* Agassiz, 1838; Amadori et al.: p. 4, figs. 2D-D^III^, 2L-L^III^, 2B, B^I^, 2M, M^I^).

v2024 *P. decurrens*; Vullo et al.; p. 3, fig. 2d.

Additional References

Agassiz, J.L.R. (1835). Rapport sur les poissons fossiles découverts depuis la publication de la troisième livraison. In J.L.R. Agassiz (Ed.), *Feuilleton additionel sur les Recherches sur les poissons fossiles, Quatrième livraison* (pp. 39-64). Neuchâtel: Imprimerie de Petitpierre et Prince.

Agassiz, J.L.R. (1838). *Recherches sur les poissons fossiles, 11th livraison.* Neuchâtel: Petitpierre (text) and H. Nicolet (atlas), 3, [73]-140, pl. 1a, 8a, 8b, 15, 17, 19, 20, 24, 25b, 30-35, 39, 40; Feuilleton additionnel, 107-116.

Agassiz, J.L.R. (1839). *Recherches sur les poissons fossiles, 10th and 12th livraisons.* Neuchâtel: Petitpierre (text) and H. Nicolet (atlas), 3, 141-156, pl. 9, 23, 25, 30a.

Alvarado-Ortega, J., Garibay-Romero, L.M., Blanco-Piñón, A., González-Barba, G., Vega-Vera, F. J., & Centeno-García, E. (2006). Los peces fósiles de la Formación Mexcala (Cretácico Superior) en el estado de Guerrero, México. *Revista Brasileira de Paleontologia, 9*(3), 261-272.

Amadori, M., Amalfitano J., Giusberti, L. Fornaciari, E., & Carnevale, G. (2019). Resti inediti di *Ptychodus* Agassiz, 1834 (Ptychodontidae, Chondrichthyes) conservati presso il Museo Civico di Rovereto (Trento). *Annali del Museo Civico di Rovereto, 34*, 221-247.

Amadori, M., Solonin, S.V., Vodorezov, A.V., Shell, R., Niedźwiedzki, R., & Kriwet, J. (2022). The extinct shark, *Ptychodus* (Elasmobranchii, Ptychodontidae) in the Upper Cretaceous of central-western Russia—The road to easternmost peri-Tethyan seas. *Journal of Vertebrate Paleontology, 42*(2), e2162909.

Amadori, M., Kovalchuk, O., Barkaszi, Z., Giusberti, L., Kindlimann, R., & Kriwet, J. (2023). A diverse assemblage of *Ptychodus* species (Elasmobranchii: Ptychodontidae) from the Upper Cretaceous of Ukraine, with comments on possible diversification drivers during the Cenomanian. *Cretaceous Research, 151*, 105659.

Amalfitano, J., Giusberti, L., Fornaciari, E., & Carnevale, G. (2020). Upper Cenomanian Fishes from the Bonarelli Level (OAE2) of northeastern Italy. *Rivista Italiana di Paleontologia e Stratigrafia, 126*(2), 261-314.

Antunes, M.T., & Cappetta, H. (2002). Sélaciens du Crétacé (Albien–Maastrichtien) d'Angola. *Palaeontographica Abteilung A, 264*, 85-146.

Astolfi, G., & Colombara, F. (2003). *La geologia dei Colli Euganei*. Padova: Edizioni Canova.

Astolfi, G., & Colombara, F. (1994). *Cava Bomba a Cinto Euganeo. La fornace - Il Museo Geopalentologico. Tesori del Veneto. Musei naturalistici 1*. Treviso: Editoriale Programma.

Berg, L.S. (1958). *System der Rezenten und Fossilen Fischartigen und Fische*. Berlin: Veb Deutscher Verlag der Wissenschaften.

Biryukov, A.V. (2018). On the Stratigraphic Significance of Elasmobranchs (Chondrichtyes, Elasmobranchii) in the Cenomanian of the Volga River Basin (Right Bank). *Series: Earth Sciences, 18*(1), 27-40. [In Russian]

Bravo-Cuevas, V.M., González-Rodríguez, K.A., Esquivel-Macías, C., & Fielitz, C. (2009). Advances on stratigraphy and paleontology of the Muhi Quarry from the Mid-Cretaceous (Albian-Cenomanian) of Hidalgo, central Mexico. *Boletín de la Sociedad Geológica Mexicana, 61*(2), 155-165.

Brignon, A. (2015). Senior synonyms of *Ptychodus latissimus* Agassiz, 1835 and *Ptychodus* *mammillaris* Agassiz, 1835 (Elasmobranchii) based on teeth from the Bohemian Cretaceous Basin (the Czech Republic). *Acta Musei Nationalis Pragae, Series B-Historia Naturalis, 71*(1-2), 5-14.

Brignon, A. (2020). Une fenêtre ouverte sur la collection paléontologique Louis Boutillier (1816-1911): aperçu des vertébrés mésozoïques. *Revue de paléobiologie, 39*(2), 421-466.

Brignon, A. (2019). *Le Diodon devenu requin. L’histoire des premiéres découvertes du genre* Ptychodus *(Chondrichthyes)*. Bourg-la-Reine : Édité par l’auteur.

Brückmann, F.E. (1752). Observatio XXIII. Dn. D. Franc. Ernest. Brückmanni. Petrefactum singulare & curiosum, dentem seu palatum piscis Ostracionis referens. In F.E. Brückmann (Ed.), *Acta Physico-Medica Academiǣ Cǣsareǣ Leopoldino - Carolinǣ Naturǣ Curiosorum Exhibentia Ephemerides Sive Observationes Historias et Experimenta a Celeberrimis Germaniǣ et Exterarum Regionum Viris Habita et Comunicata Singulari Studio Collecta* (pp. 116-120). Norimbergǣ, Impenfis B. W. M. Endteri Consortium, et Vid. B. Engelbrechti. Typis Fleischmannianis, 9.

Canestrelli, G. (1910). Denti di *Ptychodus* Agass nel Terziario dell’Appennino Tosco-Emiliano. *Atti della Società Toscana di Scienze Naturali residente in Pisa, 26*, 102-119.

Capasso, L. (2018). Implicazioni della presenza di *Ptychodus decurrens* Agassiz 1839 (Elasmobranchi, †Ptychodontidae) nel cretaceo Sup del Passo del Furlo, Italia Centrale. *Thalassia Salentina, 40*, 41-48.

Cappetta, H. (2012). *Handbook of Paleoichthyology, vol. 3E. Chondrichthyes. Mesozoic and Cenozoic Elasmobranchii: teeth*. München: Verlag Dr. Friedrich Pfeil.

Cappetta, H., Case, G.R., & Kriwet, J. (1999). Additions aux faunes de sélaciens du Crétacé du Texas (Albien supérieur-Campanien}. *Palaeo lchthyologica 9*, 5-111.

Carrillo-Briceño, J.D. (2009). Presencia del genero ‘*Ptychodus*’ (Elasmobrachii: Ptychodontidae), en el Cretácico Superior de los Andes de Trujillo, Venezuela. *Geominas, 37* (50), 207-210.

Casier, E. (1953). Origine des ptychodontes. *Memoires de l'Institut Royal des Sciences Naturelles de Belgique, 2*(49), 1-51.

Cicimurri, D.J. (2001). Cretaceous elasmobranchs of the Greenhorn Formation (Middle Cenomanian-Middle Turonian), western South Dakota. *Washington: Geologic Resources Division Technical Report, US National Park Service. Proceedings of the Sixth Fossil Resource Conference*, 27-43.

Cumbaa, S.L., Schröder-Adams, C., Day, R.G., & Phillips, A.J. (2006). Cenomanian bonebed faunas from the northeastern margin, Western Interior Seaway, Canada. *Bulletin of New Mexico Museum of Natural History and Science, 35*, 139-155.

Cumbaa, S.L., Shimada, K., & Cook, T.D. (2010). Mid-Cenomanian vertebrate faunas of the Western Interior Seaway of North America and their evolutionary, paleobiogeographical, and paleoecological implications. *Palaeogeography, Palaeoclimatology, Palaeoecology, 295*(1-2), 199-214.

Cuny, G. (2008). Mesozoic hybodont sharks from Asia and their relationships to the genus *Ptychodus*. *Acta Geologica Polonica, 58*(2), 211-216.

Davis, J.W. (1890). On The Fossil Fish of the Cretaceous Formations of Scandinavia. *The Scientific Transactions of the Royal Dublin Society, 4*(2), 363-434.

D’Erasmo, G. (1922). Catalogo dei pesci fossili delle Tre Venezie. *Memorie dell'Istituto Geologico della Regia Università di Padova, 6*, 1-181.

Dibley, G.E. (1911). On the teeth of *Ptychodus* and their distribution in the English Chalk. *Quarterly Journal of the Geological Society of London, 67*, 263-277.

Diedrich, C.G. (2012). Stomach and Gastrointestinal Tract Contents In Late Cenomanian (Upper Cretaceous) Teleosts From black shales of Germany and Analysis of Fish Mortality and Food Chains in the Upwelling-Influenced Pre-North Sea basin of Europe. *Vertebrate Coprolites: Bulletin 57*, 241-254.

Diedrich, C.G. (2013). Facies related phylostratigraphy of the benthic neoselachian *Ptychodus* from the Late Cretaceous (Cenomanian/Turonian) of the Pre–North Sea Basin of Europe. *Cretaceous Research, 41*, 17-30.

Dixon, F. (1850). *The Geology and Fossils the Tertiary and Cretaceous Formations of Sussex, London: Longman, Brown, Green, and Longmans*. London: Printed by Richard and John Edward Taylor, Red Lion Court.

Dutheil, D.B., & Ackermann, E. (2004). Présence du genre *Ptychodus* (Elasmobranchii: Ptychodontidae) dans le Cénomanien supérieur du Djebel Tselfat (Maroc). *Colloque à la mémoire de feu Anne Faure-Muret: Evolution des connaissances de la Géologie du Maroc du temps des pionniers à jours*, 1-2.

Fischer, J., Kogan, I., Popov, E., Janetschke, N., & Licht, M. (2017). The Late Cretaceous chondrichthyan fauna of the Elbtal Group (Saxony, Germany). *Research & Knowledge, 3*, 13-17.

Fritsch, A. (1878). *Die reptilien und fische der böhmischen kreideformation*. Prag: Verlag des Verfassers. In Commission bei Fr. Řivnáč.

Gallardo, C., Shimada, K., & Schumacher, B.A. (2013). A new Late Cretaceous marine vertebrate assemblage from the Lincoln Limestone Member of the Greenhorn Limestone in southeastern Colorado. *Transactions of the Kansas Academy of Science, 115*(3-4), 107-116.

Geinitz, H.B. (1875). Das Elbtalgebirge in Sachsen. VII. Classe. Fische. Placoiden. Körnschupper, Knorpelfische. In W. Dunker, & K.A. Zittel (Eds.), *Palaeontographica, Beitrage zur Naturgeschichte der vorwelt. Zwanzigster Band. Ester Theil* (pp. 277-310). Cassel: Verlag von Theodor Fischer.

Gervais, P. (1852). *Zoologie et paléontologie françaises (animaux vertébrés): ou nouvelles recherches sur les animaux vivants et fossiles de la France*. Paris: Arthus Bertrand.

González-Rodríguez, K.A., & Fielitz, C. (2009). Los peces fósiles. In K.A. González-Rodríguez, C. Cuevas-Cardona, J.M. Castillo-Cerón (Eds.), *Los fósiles del estado de Hidalgo* (pp. 65-77). Pachuca: Universidad Autónoma del Estado de Hidalgo.

Guinot, G., Underwood, C.J., Cappetta, H., & Ward, D.J. (2013). Sharks (Elasmobranchii: Euselachii) from the Late Cretaceous of France and the UK. *Journal of Systematic Palaeontology, 11*(6), 589-671.

Hamm, S.A. (2009). New data on the occurrence and distribution of Ptychodus from the Upper Cretaceous (Coniacian-Santonian) of Texas. *Dallas Paleontological Society Occasional Papers, 8*, 48-77.

Hamm, S.A. (2020). *Stratigraphic, Geographic and Paleoecological Distribution of Late Cretaceous Shark Genus Ptychodus Western Interior Seaway, North America*. Albuquerque: New Mexico Museum of Natural History and Science, Bulletin 81.

Herman, J. (1977). Les Sélaciens des terrains néocrétacés et paléocènes de Belgique et des contrées limitrophes. Eléments d'une biostratigraphie intercontinentale. *Mémoires pour servir à l’explication des Cartes géologiques et miniéres de la Belgique, 15*, 1-450.

Hoch, E. (1992). First Greenland record of the shark genus *Ptychodus* and the biogeographic significance of its fossil assemblage. *Palaeogeography, Palaeoclimatology, Palaeoecology, 92*, 277-281.

Hoffman, B.L., Hageman, S.A., & Claycomb, G.D. (2016). Scanning electron microscope examination of the dental enameloid of the Cretaceous durophagous shark Ptychodus supports neoselachian classification. *Journal of Paleontology, 90*, 741-762.

Hoyez B., Girard J., & Cottard N. (2020). *Le Cénomanien du littoral normand entre la Valleuse d’Antifer et le Cap de La Hève*. Carnets de Géologie.

Jacob, C. (1907). Etudes Paléontologiques Et Stratjgraphïques Sur La Partie Moyenne Des Terrains Crétacés Dans Les Alpes Françaises Et Les Régions Voisines. *Travaux du Laboratoire de Géologie de Grenoble, 8*(2), 280-590.

Jagt-Yazykova, E.A., & Jagt, J.W. (2015). Stratigraphy and faunal content of Turonian strata in the Opole area, southwest Poland. *13th Annual Meeting of the European Association of Vertebrate Palaeontologists Opole, Poland, 8-12 July 2015 - Field guide*, 28-35.

Kauffman, E.G. (1972). *Ptychodus* predation upon a Cretaceous *Inoceramus*. *Palaeontology, 15*(3), 439-444.

Kiprijanoff, V.A. (1852). Fisch-Ueberreste im Kurskischen eisenhaltigen Sandsteine. *Bulletin de la Société impériale des naturalistes de Moscou, 25*(4), 483-495.

Leriche, M. (1902). Révision de la faune ichthyologique des terrains crétacés du Nord de la France. *Annales de la Société Géologique du Nord 31*, 87-154.

Leriche, M. (1906). Contribution à l’étude des poissons fossiles du Nord de la France et des régions voisines. *Annales de la Société Géologique du Nord 35*, 338-357.

Limanowski, M. (1909). Sur La Tectonique Des Monts Péloritains Dans Les Environs De Taormina (Sicile). *Bulletin De La Société Vaudoise Des Sciences Naturelles, 45*(165), 1-64.

Longbottom, A.E., & Patterson, C. (1987). Fishes. In E. Owen, A.B. Smith (Eds.), *Fossils of the Chalk* (pp. 238-265). Palaeontological Association.

López-Horgue, M.Á., Bermúdez-Rochas, D.D., & Poyato-Ariza, F.J. (2018). El registro de peces cretácicos de los Pirineos occidentales: medio ambiente y paleobiogeografía. In A.B. Kortabitarte, A.G. Olivencia, & X.P. Suberbiola (Eds.), *Registro fósil de los Pirineos occidentales: bienes de interés paleontológico y geológico. Proyección social* (pp. 63-69). Vitoria-Gasteiz: Gobierno Vasco = Eusko Jaurlaritza, Servicio Central de Publicaciones = Argitalpen Zerbitzu Nagusia.

Lucas, S.G., & Spielmann, J.A. (2009). Low diversity selachian assemblage from the Upper Cretaceous Greenhorn Limestone. *New Mexico: New Mexico Geological Society, Guidebook, 60*, 311-314.

Marcinowski R., & Radwanski A. (1983). The Mid-Cretaceous transgression onto the Central Polish Uplands (marginal part of the Central European Basin). *Zitteliana, 10*, 65-95.

Mendiola, C. (2004). Primera cita española del género *Ptychodus* Agassiz 1839 (Chondrichthyes, Euselachii). *Revista de la Societat Paleontoloqica d’Elx, 13*, 1-11.

Müller, A. (2008). Ein artikulierter Fund von *Ptychodus* aus dem Obercenoman von Westfalen. *Geologie und Paläontologie in Westfalen, 70*, 55-63.

Owen, R. (1845). *Odontography, or, a Treatise on the Comparative Anatomy of the Teeth, their Physiological Relations, Mode of Development, and Microscopic Structure, in* *the Vertebrate Animals, Atlas, vol. 2*. London: Hippolyte Bailliere.

Pantanelli, D. (1905). Ancora sui resti di *Ptychodus* nell’Appennino Emiliano. *Atti della Società dei Naturalisti e Matematici di Modena, Coi Tipi di G. T. Vincenzi e Nipoti, Modena, 4*(7), 36-37.

Parkinson, J. (1811). *Organic remains of a former world, an examination of the mineralized remains of the vegetables and animals of the antediluvian world; generally termed extraneous fossils, vol. 3*. London: Printed by Whittingham and Rowland.

Passy, A. (1832). *Description géologique du département de la Seine–Inférieure*. Rouen: Imprimerie de Nicétas Periaux.

Patterson, C. (1966). British wealden sharks. *Bulletin of the British Museum (Natural History), 11*, 281-350.

Priem, F. (1912). Sur des Poissons des terrains secondaires Du Sud de la France. *Bulletin De La Société Géologique De France, 4*(12), 250-271.

Quenstedt, F.A. (1885). *Handbuch der Petrefaktenkunde. Dritte umgearbeitete und vermehrte auflage*. Tübingen: Verlag Der H. Laupp’schen Buchhandlung.

Radwański, A., & Marcinowski, R. (1996). Elasmobranch teeth from the mid-Cretaceous sequence of the Mangyshlak Mountains, Western Kazakhstan. *Acta Geologica Polonica, 46*(1-2), 165-169.

Reinhart, R.H. (1951). A new shark of the family Ptychodontidae from South America (Vol. 28). University of California Publications. *Bulletin of the Department of Geological Sciences, 28*(8), 195-202.

Reuss, A. (1845). *Die Versteinerungen Der Bohmischen Kreideformation*. Stuttgart: E. Schweizerbart’ Sche Verlagsbüchhandlung Und Druckerel.

Rindone, A. (2008). L’ittiofauna cretacia della Sicilia Nord-Orientale. Nota preliminare. *Atti del Museo Civico di Storia Naturale di Trieste, 53*, 49-66.

Rogovich, A.S. (1861). On fossil fishes of provinces of the Kiev educational district. Placoidei and Ganoidei. *Trudy komissii, vysochayshe utverzhdennoy pri Imperatorskom universitete Sv. Vladimira, dlya opisaniya guberniy Kievskogo uchebnogo okruga*, *4*(1), 1-87. [Russian]

Sacco, F. (1905). Les formations ophitifères du Crétacé. *Bulletin de la Societe Belge de Geologie de Paleontologie et d’Hydrologie, 19*, 247-256.

Seguenza, L. (1900). I vertebrati fossili della Provincia di Messina. Parte I. Pesci. *Bollettino della Società Geologica Italiana, 19*, 443-518.

Shimada, K., Rigsby, C.K., & Kim, S.H. (2009). Partial skull of Late Cretaceous durophagous shark, *Ptychodus occidentalis* (Elasmobranchii: Ptychodontidae), from Nebraska, USA. *Journal of Vertebrate Paleontology 29*(2), 336-349.

Shimada, K., Schumacher, B.A., Parkin, J.A., & Palermo, J.M. (2006). Fossil marine vertebrates from the lowermost Greenhorn Limestone (Upper Cretaceous: middle Cenomanian) in southeastern Colorado. *Journal of Paleontology, 80*(sp63), 1-45.

Siverson, M. (1999). A new large lamniform shark from the uppermost Gearle Siltstone (Cenomanian, Late Cretaceous) of Western Australia. *Earth and Environmental Science Transactions of the Royal Society of Edinburgh, 90*(1), 49-66.

Sorbini, L. (1976). L’ittiofauna cretacea di Cinto Euganeo (Padova - Nord Italia). *Bollettino del Museo Civico di Storia Naturale di Verona, 3*, 479-567.

Squinabol, S. (1904). Radiolarie cretacee degli Euganei. *Atti e Memorie della Regia Accademia di Scienze Lettere ed Arti in Padova, 20*, 171-244.

Underwood, C.J., & Cumbaa, S.L. (2010). Chondrichthyans from a Cenomanian (Late Cretaceous) bonebed, Saskatchewan, Canada. *Palaeontology, 53*(4), 903-944.

Verma, O. (2015). Cretaceous vertebrate fauna of the Cauvery Basin, southern India: Palaeodiversity and palaeobiogeographic implications. *Palaeogeography, Palaeoclimatology, Palaeoecology, 431*, 53-67.

Verma, O., Prasad, G.V., Goswami, A., & Parmar, V. (2012). *Ptychodus* *decurrens* Agassiz (Elasmobranchii: Ptychodontidae) from the Upper Cretaceous of India. *Cretaceous Research, 33*(1), 183-188.

Vullo, R., & Courville, P. (2014). Fish remains (Elasmobranchii, Actinopterygii) from the Late Cretaceous of the Benue Trough, Nigeria. *Journal of African Earth Sciences, 97*, 194-206.

Vullo, R., Villalobos-Segura, E., Amadori, M., Kriwet, J., Frey, E., González, M.A.G., Gutiérrez J.M.P., Ifrim C., Stinnesbeck E.S., & Stinnesbeck, W. (2024). Exceptionally preserved shark fossils from Mexico elucidate the long-standing enigma of the Cretaceous elasmobranch *Ptychodus*. *Proceedings of the Royal Society B, 291*(2021), 20240262.

Welton, B.J., & Farish, R.F. (1993). *The collector's guide to fossil sharks and rays from the Cretaceous of Texas*. Lewisville: Before time.

Williamson, T.E., Kirkland, J.I., & Lucas, S.G. (1993). Selachians from the Greenhorn cyclothem (“middle” Cretaceous: Cenomanian–Turonian), Black Mesa, Arizona, and the paleogeographic distribution of Late Cretaceous selachians. *Journal of Paleontology, 67*(3), 447-474.

Williamson, T.E., Lucas, S.G., & Kirkland, J.I. (1991). The Cretaceous Elasmobranch *Ptychodus decurrens* Agassiz from North America. *Geobios, 24*, 595-599.

Woodward, A.S. (1887). On the dentition and affinities of the selachian genus *Ptychodus*, Agassiz. *Quarterly Journal of the Geological Society of London, 43*(1-4), 121-131.

Woodward, A.S. (1889). *Catalogue of the fossil Fishes in the British Museum, part 1 containing the Elasmobranchi*. London: British Museum (Natural History).

Woodward, A.S. (1893). Notes on the sharks’ teeth from British Cretaceous formations. *Proceedings of the Geologists’ Association, 13*(6), 190-200.

Woodward, A.S. (1904). On the Jaws of *Ptychodus* from the Chalk. *Quarterly Journal of the Geological Society of London, 60*, 133-136.

Woodward, A.S. (1912). The fossil fishes of the English Chalk, part 7. *Monograph of the Palaeontographical Society, London, 65*, 225-264.

Zareczny, S. (1878). O srednich warstwach krίdowych w krakowskiem okregu. *Akademija umiejetno i, Krakow. Sprawozdanie komisyi fizyjograficznej, 10*, 180-216. [In Polish]

Zittel, K.A., von (1890).  Palaeozoologie: Vertebrata (Pisces, Amphibia, Reptilia, Aves), Abtheilung 1, Band 3. In W. Schimper, & A. Schenk (Eds.), *Handbuch Der Palaeontologie* (pp. 1-900). Munchen und Leipzig: Druck und Verlag von R. Oldenbourg.

Zittel, K.A., von (1902).  *Text-Book of Palaeontology. Vertebrata, vol. 2*. New York: The Macmillan Company.
